# Syphilis epidemic among men who have sex with men: A global systematic review and meta-analysis of prevalence, incidence, and associated factors

**DOI:** 10.7189/jogh.14.04004

**Published:** 2024-01-19

**Authors:** Yang Zheng, Kangli Ye, Meike Ying, Ying He, Qi Yu, Lei Lan, Wenzhi Xu

**Affiliations:** 1 Department of General Practice, The First Affiliated Hospital, School of Medicine, Zhejiang University, Hangzhou, Zhejiang, China; 2 Department of Allergy, The First Affiliated Hospital, School of Medicine, Zhejiang University, Hangzhou, Zhejiang, China; 3 Department of Endodontics, Stomatology Hospital, School of Stomatology, School of Medicine, Zhejiang University, Hangzhou, Zhejiang, China; 4 State Key Laboratory for Diagnosis and Treatment of Infectious Diseases, National Clinical Research Center for Infectious Diseases, Collaborative Innovation Center for Diagnosis and Treatment of Infectious Diseases, The First Affiliated Hospital, School of Medicine, Zhejiang University, Hangzhou, Zhejiang, China; 5 Department of Obstetrics and Gynecology, Sir Run Run Shaw Hospital, School of Medicine, Zhejiang University, Hangzhou, Zhejiang, China; 6 Key Laboratory of Reproductive Dysfunction Management of Zhejiang Province, School of Medicine, Zhejiang University, Hangzhou, Zhejiang, China

## Abstract

**Background:**

We aimed to estimate the prevalence and incidence of syphilis at global, regional and national levels for human immunodeficiency virus (HIV)-positive and HIV-negative men who have sex with men (MSM) and explore the association between demographic and social behavioural factors and syphilis infection.

**Methods:**

We searched PubMed, Embase, and the Cochrane Library from 1 January 2012 to 31 December 2022 for studies of reported crude syphilis prevalence or incidence in MSM or with sufficient data to calculate prevalence or incidence rate in MSM.

**Results:**

We included 376 articles reporting on 409 records from 62 countries to calculate syphilis prevalence and incidence in MSM. The pooled prevalence of syphilis in MSM was 10.4%, with substantial differences between countries and regions. Syphilis prevalence was substantially higher in HIV-positive than in HIV-negative MSM. The pooled incidence of syphilis in MSM was 76.4 per 1000 person-years. Older age, lower education, nitrite or recreational drug use, group sex, and multiple sexual partners were identified as risk factors for syphilis infection.

**Conclusions:**

A disproportionate geographic pattern of syphilis infection in MSM and significant threats of syphilis infection were revealed. The ‘hidden risk’ in specific regions and the inadequately elucidated drivers of high-risk behaviours, need to be fully acknowledged and addressed.

**Registration:**

PROSPERO: CRD42023422218.

Syphilis is one of the most common sexually transmitted infections globally, with an estimated 7.1 million newly infected cases per annum in 2020, according to the World Health Organization (WHO) [1]. Within the last 20 years, a substantially increasing trend of syphilis incidence was observed in most industrial countries [2,3]. Although easily curable, the infection can produce serious problems without timely recognition and treatment, considering that primary symptoms are not easily detectable or might not even appear at all. The WHO has been dedicated to strategies of stopping transmission and spreading and expanding available and affordable treatment to vulnerable populations [4].

Men who have sex with men (MSM) are at a higher risk of syphilis, particularly those who are concurrently infected with human immunodeficiency virus (HIV). This increased vulnerability is related to several factors, including a higher number of sexual partners, engaging in high-risk sexual behaviours, and the potential use of recreational drugs [5]. The rising number of syphilis cases among the population of MSM has already caused significant concern. Moreover, some syphilis cases can also be transmitted from MSM individuals to uninfected females in the general population through bisexual encounters [6]. Given these factors, it is crucial for policymakers to prioritise actions aimed at reducing syphilis transmission within the high-burden MSM population as part of comprehensive sexually transmitted infection (STI) control efforts.

Currently, only a few studies with regional-level quantitative reviews [7], non-MSM study populations [8], and non-updated original data [9] explored the syphilis burden solely in MSM. A recent global quantitative review focused on overall syphilis prevalence with limited subgroup analyses, yet it did not examine sufficient demographic and social behavioural factors and incidence rate estimation or include the role of HIV co-infection [10,11]. Understanding the prevalence and incidence of syphilis in MSM and the association with demographic, social behavioural, and HIV-coinfected factors is crucial for comprehensive prevention in practice. To date, such studies have not been fully examined at a global level for MSM. Therefore, we aimed to assess the prevalence and incidence of syphilis at the global, regional, and national levels in the population of MSM and to explore the effects of demographic, social behavioural factors, and HIV status on the prevalence of syphilis.

## METHODS

### Literature search and study inclusion

We conducted this systematic review and meta-analysis per the PRISMA guidelines and registered the protocol in PROSPERO (CRD42023422218). We comprehensively searched PubMed, Embase, and the Cochrane Library using a combination of MeSH and free text terms between 1 January 2012 and 31 December 2022. We also hand-searched HIV/STI-related conference databases (e.g. the International Acquired Immunodeficiency Syndrome (AIDS) Conference, the STI&HIV World Congress, the European Conference on Sexually Transmitted Infections) (Text S1 in the **Online Supplementary Document**). Articles and citations were managed in EndNote X9 (Clarivate Analytics, Philadelphia, USA)

### Inclusion and exclusion criteria

We included studies on MSM, defined as those who were assigned as male at birth and had insertive or receptive oral/anal sexual intercourse with another man during their lifetime, regardless of their HIV status; studies that reported crude syphilis prevalence or incidence in MSM or with sufficient data (e.g. number of positive syphilis cases and sample size or follow-up person-years) to calculate prevalence or incidence rate in MSM; and studies that estimated syphilis infection using laboratory-derived methods. We excluded studies on transgender populations or populations with possible severe selection bias (e.g. estimation of syphilis infection rate among oral human papillomavirus/anal chlamydia positive MSM individuals); reported only on site-specific syphilis cases (e.g. rectal or syphilitic hepatitis); had a sample size <50; contained duplicated data from another study; were designed as a case report/series or review; or if they did not have original data (e.g. modelling studies). No language restrictions were applied.

### Data extraction

Four authors (KY, MY, YH, and QY) initially screened the titles and abstracts of all articles to exclude those that did not contain syphilis infection rates or suitable data for calculation. Two authors (YZ and WX) then screened the remaining full-text articles, cross-checking for validity and accuracy. Disagreements were resolved through consensus by all authors.

We extracted the following relevant information from eligible studies into a standardised form in Microsoft Excel 2016 (Microsoft, Washington DC, USA): basic information (title, first author, journal name, publication year, study period, country and cities, sampling method, and study design), demographic and epidemiologic information (sample size or follow-up person year, mean or median age, education level, and economic level), social and behaviour information (condom use, risky sexual behaviour, recreational drug use, commercial sex, sexual debut, venue, and partner number), testing method, testing result, age-specific testing result, type-specific testing result, and HIV-coinfection status.

### Risk of bias assessment

We assessed the quality of included studies with the Newcastle-Ottawa Scale (NOS) for cohorts and case-control studies and a modified NOS for cross-sectional studies [12,13]. The NOS assesses three domains of study methodology, including study participant selection (0–4 points), confounder adjustment (0–2), and outcome indicator determination (0–3). The sum of the points of the three domains represented the overall bias risk of each study (Tables S1–2 in the **Online Supplementary Document**). Studies with an NOS score of 7–9 points were defined as high quality, while those with <7 were deemed low or medium quality. We assessed publication bias by Egger’s regression-based test and visually inspecting the funnel plot. We also performed a leave-one-out sensitivity analysis to assess the influence of each individual study on the final pooled estimates.

### Statistical analysis

We calculated the pooled prevalence and incidence of syphilis infection in MSM and their 95% confidence intervals (CI) using a random-effect model. We conducted a Freeman-Tukey double arcsine transformation of the original rate to stabilise the variance to reduce the effect of extreme values on the pooled positive rate estimate, presenting the results as ‘per cent’ for prevalence and ‘per 1000 person-years’ for incidence with corresponding 95% CIs. We assessed heterogeneity using the *I*^2^ and also conducted a subgroup analysis to estimate the prevalence of syphilis in subgroups by HIV status, age categories, education level, sexual behaviours (i.e. condom use, group sex, lubricant use, recreational drug use, commercial sex), age of sexual debut, main venue to find partner, and partner number. We further explored the association between sexual behaviours, age of sexual debut, partner number in the past three months, and syphilis infection by pooling odds ratios (ORs) using DerSimonian and Laird random-effects models. Data were analysed using R, version 4.0.0 (R Core Team, Vienna, Austria). We considered *P* < 0.05 statistically significant.

## RESULTS

**Figure 1:**
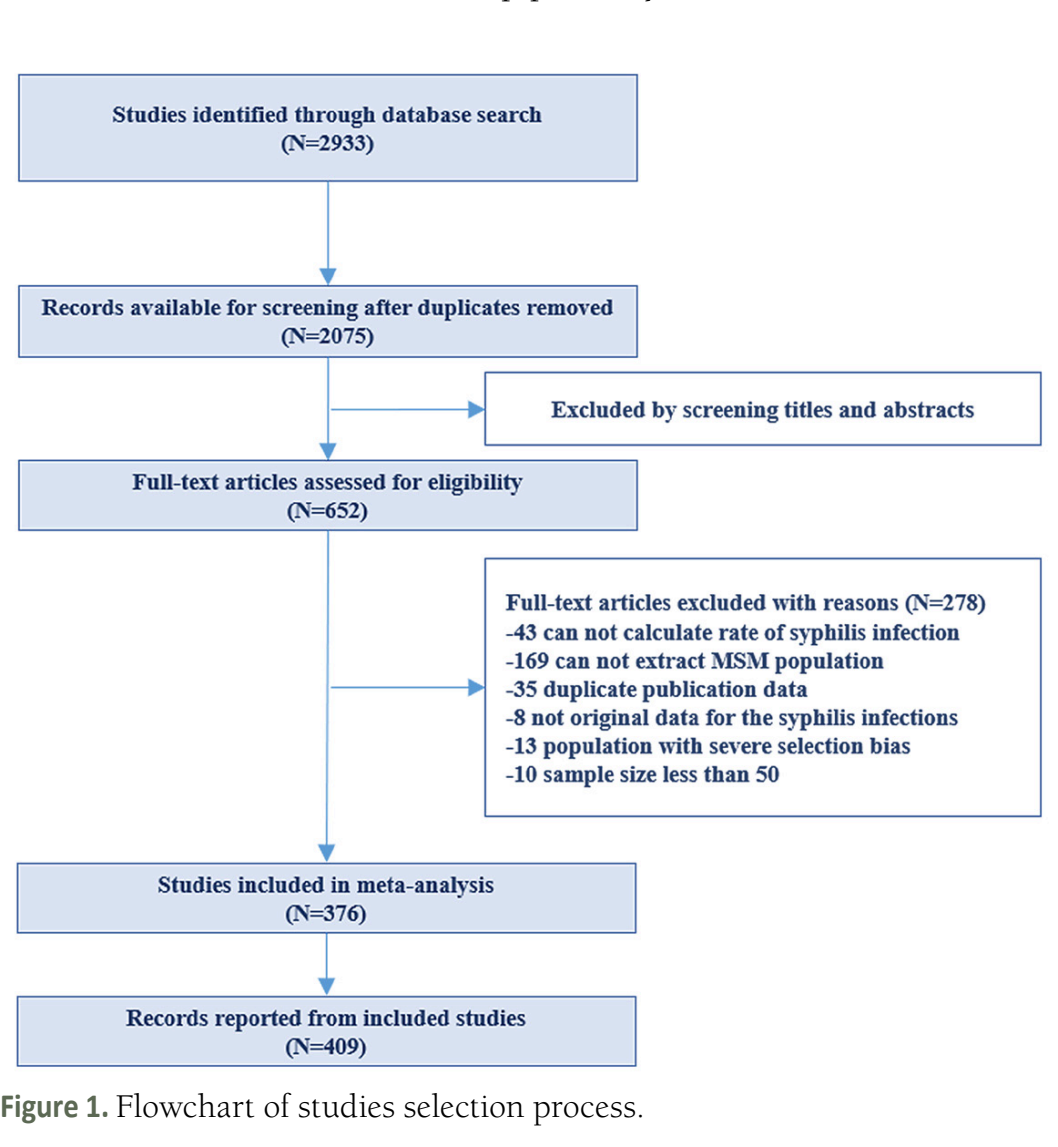
Flowchart of studies selection process.

We screened 2933 articles retrieved from the databases, with 2075 remaining following deduplication. We excluded 1423 articles during title and abstract screening, leaving 652 potentially relevant ones for full-text review. We finally included 376 articles reporting 409 records from 62 countries, with 1 529 065 participants and 294 626.8 follow-up person-years (**Figure 1**). The records were published between 2012 and 2022. The prevalence studies had between 68 and 285 018 participants, while incidence studies had between 38.3 to 112 960 follow-up person-years. All studies that mentioned specific tests used syphilis antibody tests. Most studies were cross-sectional (n = 338) and cohort (n = 71) in design. A total of 151 studies reported that there were 11 983 HIV-positive syphilis infections, while there were 57 027 HIV-negative infections in 405 639 MSM (Tables S1–2 in the **Online Supplementary Document**).

**Figure 2:**
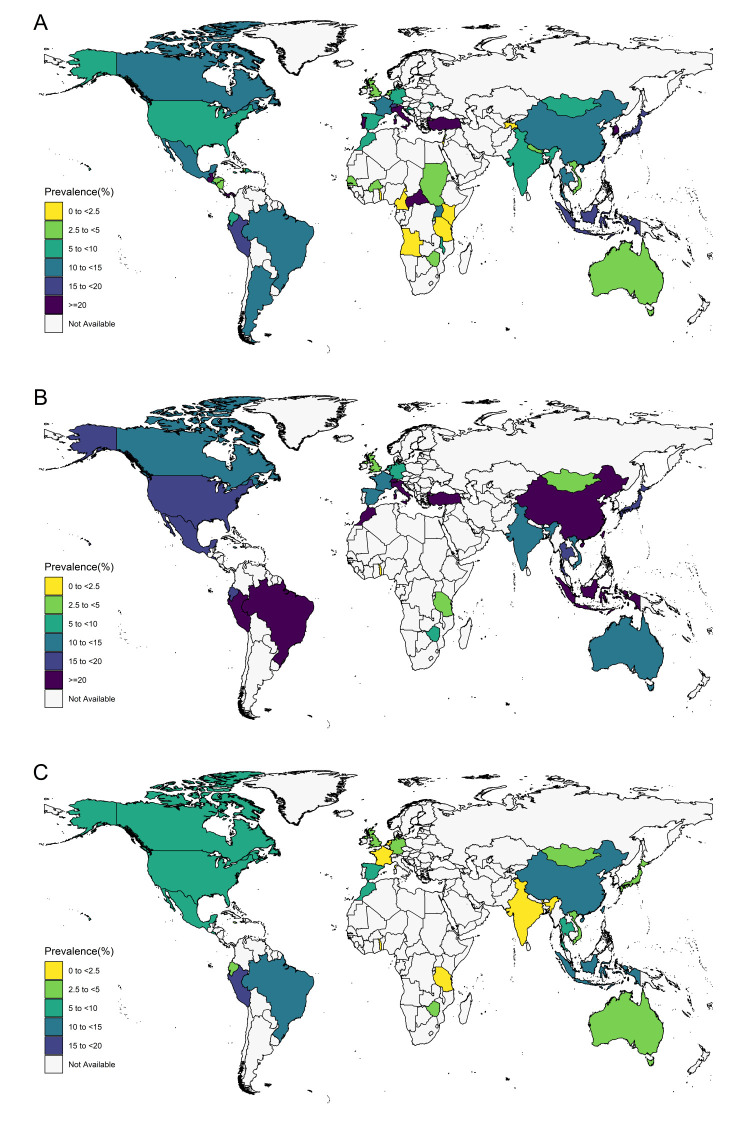
Estimated syphilis prevalence in MSM by country. **Panel A.** Syphilis prevalence in overall MSM population. **Panel B.** Syphilis prevalence in HIV-positive MSM subpopulation. **Panel C.** Syphilis prevalence in HIV-negative MSM subpopulation

The pooled prevalence of syphilis in MSM was 10.4% (95% CI = 9.7–11.1) globally, with notable variations by region and country. For example, North Africa and the Middle East had the highest reported syphilis prevalence (15.6%; 95% CI = 7.6–5.6), followed by Latin America and Caribbean (14.5%; 95% CI = 11.4–17.8) and Southeast Asia, East Asia, and Oceania (10.4%; 95% CI = 9.7–11.1). Meanwhile, sub-Saharan Africa had the lowest prevalence of all regions (2.5%; 95% CI = 1.3–4.1) (**Table 1**). Nine countries had prevalence rates of ≥20%, with the highest occurring in Portugal (42.6%; 95% CI = 35.8–49.5). Seven countries had prevalence rates of <2.5%, with the lowest occurring in Togo (0.3%; 95% CI = 0.0–2.1) (**Figure 2**). The pooled prevalence ranged from 10.3% to 10.4% in the leave-one-out sensitivity analysis, with no single study significantly influencing the overall pooled prevalence in the meta-analysis. Visual inspection of the funnel plot and Egger’s test demonstrated potentially significant publication bias (Figures S1–3 in the **Online Supplementary Document**).

**Table 1:** Estimates of syphilis prevalence and incidence by geographic region

	Prevalence			Incidence		
**Geographic region**	**Record**	**Number of participants**	**Pooled prevalence estimates, % (95% CI)**	**Record**	**Follow-up, person-years**	**Pooled incidence estimates, 1000 person year (95% CI)**
South-East Asia, East Asia, and Oceania*	166	755 908	11.8 (11.2–12.5)	22	20 877.0	84.4 (64.4–106.7)
*China*	145	726 409	12.0 (11.3–12.7)	17	13 987.9	85.7 (68.3–104.9)
*Hong Kong, SAR of China*	2	523	5.0 (1.0–11.3)			
*Indonesia*	1	2912	16.2 (14.9–17.6)	1	488.4	81.9 (59.1–108)
*Taiwan, province of China*	4	8098	18.4 (5.6–36.3)	1	665.5	199.8 (170.3–231.1)
*Thailand*	9	10 489	13.4 (8.5–19.3)	3	5735.3	46.5 (19.7–83.8)
*Vietnam*	5	6925	4.16 (1.67–7.66)			
South Asia*	10	37 602	6.2 (4.3–8.4)			
*India*	9	37 435	6.4 (4.4–8.7)			
*Nepal*	1	167	4.2 (1.6–7.9)			
North Africa and the Middle East*	6	3586	15.6 (7.6–25.6)			
*Morocco*	2	1338	9.8 (7.9–12.0)			
*Lebanon*	1	1429	3.0 (2.2–4.0)			
*Turkey*	3	819	26.61 (20.09–33.68)			
High income*	86	681 603	8.0 (6.8–9.2)	42	269 182.6	69.4 (44.7–98.9)
*Argentina*	2	722	14.1 (4.4–27.8)	1	1150.0	148.7 (128.7–169.9)
*Australia*	12	224 768	3.0 (1.7–4.6)	5	30 037.6	56.6 (22.2–105.4)
*Belgium*				1	1315.8	133 (115.2–151.9)
*Canada*	6	18 678	11.6 (4.9–20.7)	4	5839.6	49.7 (21.2–88.3)
*France*	5	5561	10.1 (1.0–26.5)	4	1808.3	149.1 (19.6–364.6)
*Germany*	6	5919	5.1 (2.2–9.1)	1	4057.0	40.7 (34.8–47)
*Israel*	1	1064	0.7 (0.3–1.3)			
*Italy*	2	260	27.8 (17.7–39.1)			
*Japan*	3	2469	16.1 (8.5–25.7)	2	2633.6	41.1 (33.6–49.4)
*Netherlands*	4	57 935	4.6 (3.4–6.1)	2	5647.7	52.9 (0.4–180.6)
*New Zealand*				1	133.3	105.1 (58–163.5)
*Portugal*	2	202	42.6 (35.8–49.5)			
*Singapore*	2	199	11.4 (2.1–26.1)	1	2477.0	90 (79.1–101.6)
*South Korea*	1	108	20.4 (13.3–28.5)			
*Spain*	4	7808	9.5 (3.4–18.2)	2	207.7	64.1 (0–267.1)
*Switzerland*	2	840	15.5 (0.0–56.0)	1	17 653.1	490 (482.6–497.4)
*UK*	5	297 447	3.39 (1.93–5.23)	2	113 073.0	30.5 (29.4–31.5)
*USA*	29	57 075	7.74 (5.86–9.84)	15	82 655.0	42.8 (31.1–56.1)
Sub-Saharan Africa*	17	8016	2.5 (1.3–4.1)	1	61.0	32.8 (0.5–96.3)
*Angola*	1	310	0.3 (0.0–1.4)			
*Burkina Faso*	2	1329	3.3 (0.1–10.7)			
*Cameroon*	1	511	0.4 (0.0–1.2)			
*Central African Republic*	1	99	21.2 (13.7–29.9)			
*Kenya*	1	519	0.8 (0.1–1.8)			
*Malawi*	1	337	5.3 (3.2–8.0)			
*Rwandan*	1	500	3.4 (2.0–5.2)			
*Senegal*	1	115	2.6 (0.3–6.5)	1	61.0	32.8 (0.5–96.3)
*South Sudan*	1	152	3.3 (0.9–6.8)			
*Tanzania*	3	1317	0.9 (0.2–2.0)			
*Togo*	2	1361	0.31 (0–2.05)			
*Uganda*	1	290	10.34 (7.08–14.13)			
*Zimbabwe*	1	1176	4.76 (3.61–6.06)			
Latin America and the Caribbean*	47	41 029	14.5 (11.4–17.8)	4	4011.5	70.7 (50.5–93.8)
*Brazil*	13	20 662	14.1 (9.2–19.8)			
*Dominican Republic*	1	1388	5.5 (4.3–6.7)			
*Ecuador*	3	1075	7.7 (4.9–11.1)			
*El Salvador*	4	2824	6.3 (3.0–10.7)			
*French Antilles*				1	2384.3	54.9 (46.1–64.5)
*Guatemala*	3	1257	28.3 (0.8–73.4)			
*Haiti*	1	520	4.2 (2.7–6.2)			
*Honduras*	1	553	4.0 (2.5–5.8)			
*Jamaica*	1	201	6.0 (3.1–9.7)			
*Mexico*	4	886	13.8 (8.1–20.6)	1	161.0	130.4 (82.4–187.2)
*Nicaragua*	1	590	3.2 (1.9–4.8)			
*Panama*	1	600	23.5 (20.2–27.0)			
*Peru*	10	9845	17.0 (10.3–24.9)	2	1466.2	63.1 (50.9–76.4)
*Puerto Rico*	1	78	29.5 (19.8–40.1)			
*Trinidad and Tobago*	3	550	35.8 (26.39–45.78)			
Central Europe, Eastern Europe, and Central Asia*	6	2421	7.4 (3.1–13.4)	2	988.6	180.3 (0.0–563.6)
*Croatia*	2	477	8.6 (4.0–14.8)			
*Mongolia*	1	1045	6.8 (4.5–9.5)	1	937.1	56.6 (42.6–72.3)
*Poland*			0 (0–0)	1	51.5	368.9 (241.5–506)
*Republic of Moldova*	1	397	6.8 (4.5–9.5)			
*Tajikistan*	1	502	2.2 (1.1–3.7)			
Total	338	1 530 165	10.4 (9.7–11.1)	71	295 120.8	76.4 (56.9–98.3)

CI – confidence interval, HIV – human immunodeficiency virus, MSM – men who have sex with men, SAR – special administrative region

*Results on global or regional level.

The pooled incidence of syphilis in MSM was 76.4 (95% CI = 56.9–98.3) per 1000 person-years globally. Almost all studies were conducted in high-income areas or Southeast Asia, East Asia, and Oceania regions (**Table 1**). In all, six countries had incidence rates of <48.1 and five had rates of ≥140.9 per 1000 person-years, respectively. Switzerland had the highest (490 per 1000 person-years; 95% CI = 482.6–497.4), and UK had the lowest syphilis incidence (30.5 per 1000 person-years; 95% CI = 29.4–31.5) (Appendix p8 in the **Online Supplementary Document**). The sensitivity analysis showed that the pooled prevalence of incidence was not affected significantly by a single study (ranged from 7.0–78.0 per 1000 person-years) . The funnel plot and Egger’s test showed no publication bias.

In the subgroup analysis of different HIV infection statuses, the pooled prevalence of syphilis was 22.2% (95% CI = 20.0–24.5) in HIV-positive MSM vs 8.8% (95% CI = 8.0–9.7) in HIV-negative MSM (**Table 2**). Across 151 studies screening 69 010 HIV-positive participants, pooled prevalence was estimated to be the highest for North Africa and the Middle East (28.1%; 95% CI = 21.3–35.5), Latin America and the Caribbean (27.8%; 95% CI = 20.7–35.6), and Southeast Asia, East Asia, and Oceania (26.0%; 95% CI = 23.3–28.8), and the lowest for sub-Saharan Africa (3.0%; 95% CI = 0.0–11.2). Across 126 studies screening 333 451 HIV-negative participants, the highest pooled prevalence estimates were given for Latin America and the Caribbean (10.6%; 95% CI = 7.2–14.5) and Southeast Asia, East Asia and Oceania (9.9%; 95% CI = 8.9–11.0), and the lowest for sub-Saharan Africa (0.8%; 95% CI = 0.0–3.2). In HIV-positive MSM, the pooled incidence of syphilis was 150.1 (95% CI = 115.4–209.7) per 1000 person-year, which was higher than the 79.5 (95% CI = 49.6–115.4) per 1000 person-years in HIV-negative MSM (Appendix pp8-9 in the **Online Supplementary Document**).

**Table 2:** Estimates of syphilis prevalence by HIV status

	HIV+ MSM			HIV− MSM		
**Geographic region**	**Record**	**Number of participants**	**Pooled prevalence estimates, % (95% CI)**	**Record**	**Number of participants**	**Pooled prevalence estimates, % (95% CI)**
South-East Asia, East Asia and Oceania*	89	26 808	26.0 (23.3–28.8)	83	206 397	9.9 (8.9–11.0)
*China*	74	21 876	27.1 (24.0–30.3)	73	193 984	10.3 (9.2–11.4)
*Hong Kong, SAR of China*	1	17	29.4 (9.7–53.6)	2	506	4.4 (0.2–12.5)
*Indonesia*	1	699	27.5 (24.2–30.8)	1	2213	12.7 (11.3–14.1)
*Taiwan, province of China*	3	1942	29.3 (14.4–46.9)	1	232	13.4 (9.3–18.1)
*Thailand*	8	1844	19.3 (10.7–29.6)	4	4351	9.6 (3.9–17.5)
*Vietnam*	2	430	13.6 (3.1–29.7)	2	5111	2.9 (0.6–6.7)
South Asia*	1	1126	12.0 (10.2–14.0)	1	10 634	2.4 (2.1–2.7)
*India*	1	1126	12.0 (10.2–14.0)	1	10 634	2.4 (2.1–2.7)
North Africa and the Middle East*	4	847	28.1 (21.3–35.5)	1	641	7.5 (5.6–9.7)
*Morocco*	1	28	39.3 (21.8–58.2)	1	641	7.5 (5.6–9.7)
*Turkey*	3	819	26.6 (20.1–33.7)			
High-income*	35	37 948	14.0 (10.6–17.8)	22	109 292	5.9 (4.8–7.2)
*Australia*	2	13 535	11.5 (5.7–19.0)	2	84 832	4.1 (3.6–4.7)
*Canada*	4	7215	13.2 (1.8–32.5)	2	8928	5.5 (3.0–8.7)
*France*	4	5333	12.3 (1.2–31.8)	1	17	0.0 (0.0–9.9)
*Germany*	4	3496	6.6 (3.0–11.4)	1	1043	3.6 (2.5–4.8)
*Italy*	1	186	32.8 (26.2–39.7)			
*Japan*	3	2177	17.4 (11.7–23.9)	1	300	3.0 (1.3–5.3)
*Netherlands*	2	835	7.6 (2.6–14.9)	1	494	2.0 (0.9–3.5)
*Singapore*	1	127	18.1 (11.9–25.3)			
*Spain*	2	710	14.2 (8.4–21.3)	1	250	8.4 (5.3–12.2)
*Switzerland*	1	112	34.8 (26.2–43.9)			
*UK*	1	30	3.3 (0.0–13.8)	1	6585	2.8 (2.4–3.2)
*USA*	10	4192	17.5 (8.0–29.6)	12	6843	8.9 (4.7–14.4)
Sub-Saharan Africa*	3	540	3.0 (0.0–11.2)	3	2184	0.8 (0.0–3.2)
*Tanzania*	1	143	2.8 (0.6–6.3)	1	503	0.6 (0.1–1.5)
*Togo*	1	149	0.0 (0.0–1.2)	1	529	0.0 (0.0–0.3)
*Zimbabwe*	1	248	9.7 (6.3–13.7)	1	1152	2.8 (1.9–3.8)
Latin America and the Caribbean*	18	1720	27.8 (20.7–35.6)	15	4115	10.6 (7.2–14.5)
*Brazil*	6	675	27.3 (13.2–43.9)	5	1144	12.8 (7.1–19.8)
*Ecuador*	2	92	16.1 (9.1–24.5)	2	692	4.6 (3.2–6.3)
*El Salvador*	2	171	17.5 (3.8–37.7)	2	1219	7.6 (2.4–15.3)
*Jamaica*	1	65	10.8 (4.2–19.6)	1	136	3.7 (1.0–7.6)
*Mexico*	1	33	18.2 (6.6–33.4)	1	158	5.1 (2.1–9.1)
*Peru*	3	134	49.2 (19.2–79.5)	4	766	18.5 (11.7–26.4)
*Trinidad and Tobago*	3	550	35.8 (26.4–45.8)			
Central Europe, Eastern Europe, and Central Asia*	1	21	4.8 (0.0–19.3)	1	188	3.7 (1.4–7.0)
*Mongolia*	1	21	4.8 (0.0–19.3)	1	188	3.7 (1.4–7.0)
Total	151	69 010	22.2 (20.0–24.5)	126	333 451	8.8 (8.0–9.7)

CI – confidence interval, HIV – human immunodeficiency virus, MSM – men who have sex with men, SAR – special administrative region

*Results at global or regional level.

**Figure 3:**
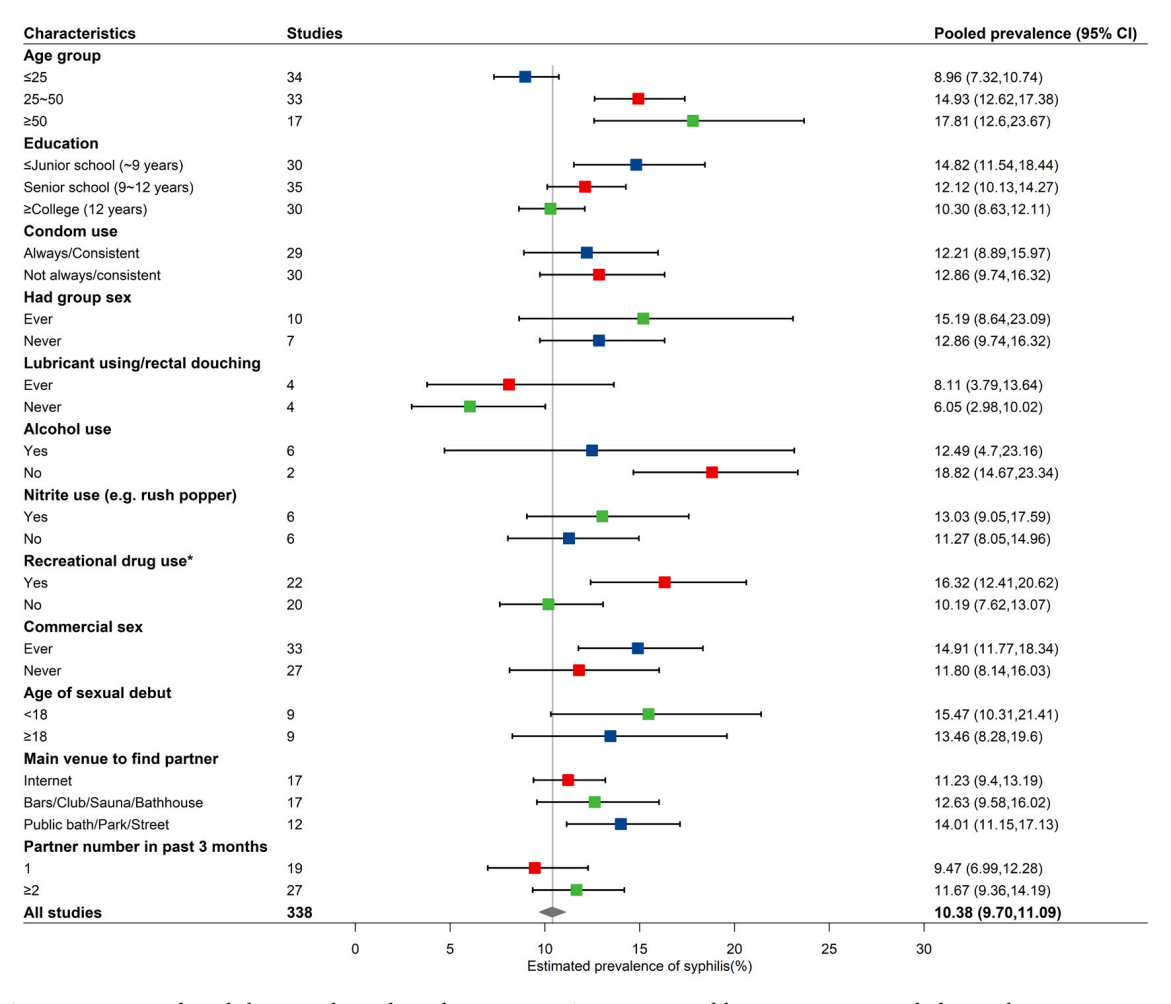
Estimated syphilis prevalence by subgroup in MSM. Points and lines represent pooled prevalence estimates and 95% confidence intervals (CIs).

In the subgroup analysis of demographic and sexual behaviour factors, the pooled syphilis prevalence was higher in the ≥50-year-old age group (17.81%; 95% CI = 12.60–23.67), the group with a ‘lower than junior school’ education level (14.82%; 95% CI = 11.54–18.44), and among individuals who frequented public bath/park/street as the main venue to find a partner (14.01%; 95% = 11.15–17.13) (**Figure 3**). MSM who ever had group sex had a significantly higher syphilis prevalence than those who never had group sex (15.19% vs 12.86%, odds ratio (OR) = 2.05; 95% CI = 1.57–2.69, *P* < 0.001). MSM who ever used lubricant or rectal douching had a significantly higher syphilis prevalence (8.11% vs 6.05%, OR = 1.27; 95% CI = 1.01–1.61, *P* = 0.044). We also observed higher syphilis prevalence for nitrite users, such as rush popper users (13.03% vs 10.19%, OR = 1.23; 95% CI = 1.05–1.44, *P* = 0.009) and other recreational drug users (16.32% vs 10.19%, OR = 1.79; 95% CI = 1.42–2.26, *P* < 0.001), than for non-nitrite users and non-recreational drug users, respectively. MSM who reported involvement in commercial sex also had a significantly higher prevalence than those who reported involvement in non-commercial sex (14.91% vs 11.80%, OR = 1.33; 95% CI = 1.04–1.70, *P* = 0.024). Syphilis prevalence was higher in MSM reporting ≥2 partners in the past three months than in studies reporting a single partner (11.67% vs 9.47%, OR = 1.34, 95% CI = 1.05–1.71, *P* = 0.019). Pooled estimates of syphilis prevalence were not different between MSM consistently using condoms and MSM not consistently using condoms (12.21% vs 12.86%, OR = 0.94; 95% CI = 0.79–1.11, *P* = 0.457), between alcohol users and non-alcohol users (12.49% vs 18.82%, OR = 0.89; 95% CI = 0.54–1.48, *P* = 0.661), or between studies reporting a mean age at sexual debut of <18 years and ≥18 years (15.47% vs 13.46%, OR = 1.16; 95% CI = 0.87–1.54, *P* = 0.323) (Figure S5 in the **Online Supplementary Document**).

## DISCUSSION

This global large-scale meta-analysis included 376 studies with 1 529 065 MSM and 294 626.8 follow-up person-years from 62 countries and provided a comprehensive overview of the syphilis prevalence and incidence in the population of MSM. We observed a geographic pattern of syphilis infection in MSM, with the highest prevalence in North Africa and the Middle East and the lowest prevalence in regions of sub-Saharan Africa; a higher prevalence in HIV-positive MSM; and distinctively higher estimates of syphilis in subgroup populations with older age, lower education, nitrite or recreational drug use, multiple sexual partners, and high-risk sexual behaviours.

In our analysis, the prevalence of syphilis was similar to a previously reported rate by Mahmud et al. (10.4% vs 9.86%) from 12 Asian countries with 129 090 MSM respondents [7], but was substantially higher than those in another global meta-analysis (7.5%; 95% CI = 7.0–8.0) from 77 countries in the MSM population between 2000 and 2020 by Tsuboi et al. [10]. The lower pooled prevalence in our study can possibly be explained by the fact that the previous global synthesised analyses excluded MSM living with HIV, injection drug users, and individuals routinely visiting clinics. Likewise, we did not include surveillance data as previous analyses did, which could have led to the lower estimation due to limited capture or incomplete coverage [14,15]. Notably, our estimation was nearly 20-fold higher than the estimated global syphilis prevalence in the general male population (0.5%; 95% CI = 0.3–0.7) and 13-fold higher than those for pregnant women (0.8%; 95% CI = 0.7–0.9) [8,16]. Due to this huge disparity, our findings still support the strategies of sustained commitment and high priority of prevention, screening, and treatment for high-risk populations.

We found a wide variation in the prevalence and incidence of syphilis in MSM among countries and regions. For example, we observed a higher prevalence and incidence in Latin America and the Caribbean, as well as North Africa and the Middle East. One likely factor behind this finding is the lack of comprehensive and competent health care systems of countries in these regions, preventing individuals from seeking high-quality health care [17,18]. Meanwhile, economic instability, poverty, and political issues may also contribute to inadequate health services and even the breakdown of the health care systems in these regions [18]. Another influential factor may be the local presence of stigma and discrimination against MSM, making them reluctant to seek health care and further contributing to the spread of syphilis [19]. Contrary to what is often assumed, we observed the lowest pooled prevalence and incidence of in sub-Sahara Africa. Although in line with the findings of Tsuboi et al. [10], this observation is difficult to explain. There is evidence to suggest that populations in sub-Saharan Africa may face a huge burden of STIs [20]; however, they may suffer from under-reported or under-recognised STIs due to stigma and discrimination, lack of access to health care and syphilis testing, limited surveillance and data collection, and reduced willingness to expose or engage in MSM sexual activity [21]. However, only 17 studies (5.03%) and 8016 participants (0.52%) included in our analysis were from sub-Saharan Africa. Overall, this problem requires a multifaceted approach that encompasses improving access to comprehensive sexual health services, reducing stigma and discrimination, and addressing economic and political factors. This ‘hidden risk’ in sub-Saharan Africa is a significant public health concern that should be fully recognised.

We observed that syphilis prevalence was significantly higher (i.e. up to five times) in HIV-positive than in HIV-negative MSM, with the disparity being most distinct in South Asia and sub-Saharan Africa. Similarly, other STIs were also detected to be significantly more prevalent in HIV-positive individuals [22]. The connection between ‘classic STIs’ and HIV was first referred to as ‘epidemiologic synergy’ in the 1990s; recently, several studies have also explored this connection and proposed several underlying mechanisms [23]. One of the widely accepted hypotheses is that HIV depresses the immune system, thereby increasing the biological susceptibility to various infectious diseases [24]. Additionally, HIV-positive patients are more likely to engage in high-risk sexual behaviours and high-prevalence networks, such that HIV and other STIs are often epidemiologically linked [25]. Although routine regular STI screening for HIV-positive individuals has been recommended and implemented in many industrial countries, it could still be challenging due to those with limited resources and weaker health care infrastructure in less developed regions [26]. Therefore, integrated public health efforts focussed on HIV/STIs need to be strengthened and tailored approaches designed that consider local contexts and resource constraints in low- and middle-income countries.

Our subgroup analysis indicated that MSM with older age, lower education, nitrite or recreational drug use, more frequent group sex, and multiple sexual partners had a higher syphilis prevalence, which aligns with previous studies. Evidence suggests that older people have been largely ignored by healthcare providers, and that they frequently reported to have low self-perception and limited knowledge because of age-related stigma [27]. This should inform medical professionals to raise awareness among and offer more specialised preventative interventions for older MSM. Notably, we identified recreational drug use and group sex as risk factors with the highest ORs. Previous results also suggested a substantial increase in HIV/STI infections among recreational drug users. According to literature, recreational drug use would initially lead to an increase in behaviours that are associated with sexually risky lifestyles, such as having multiple sex partners, participating in group sex, and engaging in unprotected intercourse and internal ejaculation without a condom, because the drug would increase sexual desire and pleasure [28]. Moreover, recreational drugs may also impair judgment and decision-making, leading to decreased use of condoms and increased likelihood of engaging in risky sexual behaviours [29]. Thereafter, recreational drug use-driven behavioural modifications brought on by the aforementioned causes are probably going to serve as mediators and raise the risk of contracting syphilis [30]. Thus, fully addressing the underlying psychological and social drivers of recreational drug use is needed to minimise harm in the MSM population [31].

Similar to the study by Tsuboi et al. [10], we attempted to estimate the prevalence and incidence of syphilis at the global and national level in MSM. We also summarised the effects of demographics, social behaviour, HIV status, and other varying factors on syphilis infection. Our findings should still be interpreted with caution due to several limitations. First, our results suffered limited generalisability and potential systematic errors because most records came from high-income areas and Southeast Asia, East Asia, and Oceania regions, and several of countries only have one or two records. Moreover, the proportion of HIV-positive participants varied by country, suggesting that more primary studies are needed, especially from areas with limited data. Second, the diverse range of studies included resulted in significant heterogeneity, which we sought to address by using a random effects model and conducting subgroup analyses by demographic and social behaviour factors and HIV status. Third, although we conducted a comprehensive literature search and strict data extraction, we still observed publication bias in the pooled prevalence rate, indicating that our search may have missed some studies, leading to possible overestimates of the actual rate. Fourth, the pooled rate in our study is the ‘reported/diagnosed’ rate, which may not reflect the ‘true/real’ rate due to factors such as diagnostic and analytical accuracy. Future studies are needed for more accurate estimations in conjunction with additional corrective techniques.

Consequently, we offer some recommendations for improving future primary studies. For accurate and unbiased estimation of syphilis infection, large-scale studies should use combined diagnostic testing of treponemal and nontreponemal tests. Likewise, more prospective cohort studies are required that go deeper into and validate the possible relationship between morbidity and variables that were assumed to be relevant, but were not disclosed in our analysis.

## CONCLUSIONS

Our findings suggest a disproportionate geographic pattern of global syphilis infection in MSM and reveal significant demographic and social behavioural threats to syphilis infection. Existing integrated public health efforts, including those focussed on improving health care services, reducing stigma and discrimination, and performing routine screening, should continue to be implemented in the context of regional cultures and resources. Additional public health issues represented by the ‘hidden risk’ in specific regions and the inadequately explored drivers of high-risk behaviours need to be fully acknowledged and addressed.

## Additional material


Online Supplementary Document
