# Supplementary material for: Syphilis epidemic among men who have sex with men: A global systematic review and meta-analysis of prevalence, incidence, and associated factors
Source: J Glob Health. 2024 Jan 19;14:04004. doi: 10.7189/jogh.14.04004 (PMC10795860; doi:10.7189/jogh.14.04004)
Supplement: Online Supplementary Document [file jogh-14-04004-s001.pdf]

Appendix to **Syphilis epidemic among men who have sex with men: A global systematic review and meta-analysis of prevalence, incidence, and associated factors**

Text

p.2. Text S1. Search strategy

Figures

p.3. Figure S1. Sensitivity analysis of syphilis prevalence study

p.5. Figure S2. Sensitivity analysis of syphilis incidence study

p.6. Figure S3. Funnel plot and publication bias

p.8. Figure S4. Estimated syphilis incidence in MSM by country

p.9. Figure S5. Associations (odds ratios) between demographic, social behavior factors and syphilis infection in different geographic regions

Tables

p.13. Table S1. Characteristics of the included prevalence studies.

p.36. Table S2. Characteristics of the included incidence studies.

p.41. Table S3. Estimates of syphilis incidence by HIV status

Text S1. Search strategy (searching date 2022-09-10)

1. Pubmed/MEDLINE

Search year: 2012-2022

Step	Searching text	Result
#1	"sexual and gender minorities"[MeSH Terms] OR "homosexuality, male"[MeSH Terms] OR "MSM"[Title/Abstract] OR "men who have sex with men"[Title/Abstract] OR "gay"[Title/Abstract]	28276
#2	"syphilis"[MeSH Terms] OR "syphilis"[Title/Abstract] OR ” Treponema pallidum [Title/Abstract]”	8848
#3	((((epidemiology study) OR (epidemiologic)) OR (prevalence)) OR(prevalent) OR (incidence)) OR (incident)	2,072,333
#4	#1 AND #2 AND #3	1207

2. Embase

Search year: 2012-2022

Step	Searching text	Result
#1	('homosexual*':ab,ti OR 'msm':ab,ti OR 'men who have sex with men':ab,ti OR 'gay':ab,ti)	49457
#2	('syphilis':ab,ti OR 'treponema pallidum':ab,ti)	35105
#3	(epidemiolog* OR 'incidence'/exp OR incidence OR incident OR 'prevalence'/exp OR prevalence OR prevalent)	4483709
#4	#1 AND #2 AND #3 AND 2012-2022:py	1647

Cochrane Library

Search year: 2012-2022

Title abstract keyword

Step	Searching text	Result
#1	homosexual* OR MSM OR "men who have sex with men" OR "gay"	2209
#2	"syphilis" OR "Treponema pallidum"	817
#3	epidemiolog* OR incidence OR incident OR prevalence OR prevalent	224050
#4	#1 AND #2 AND #3	79

Figure S1. Sensitivity analysis of syphilis prevalence study

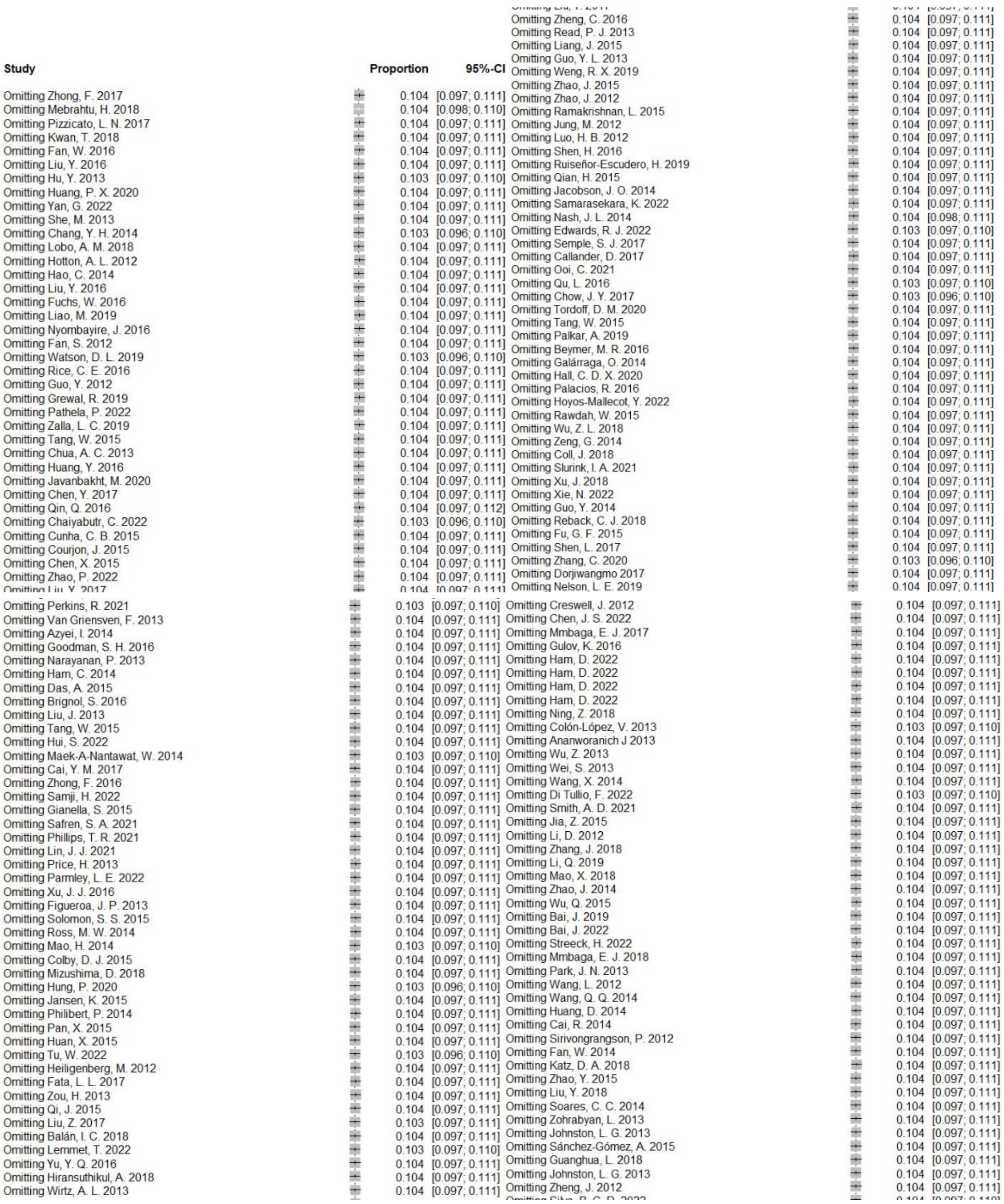

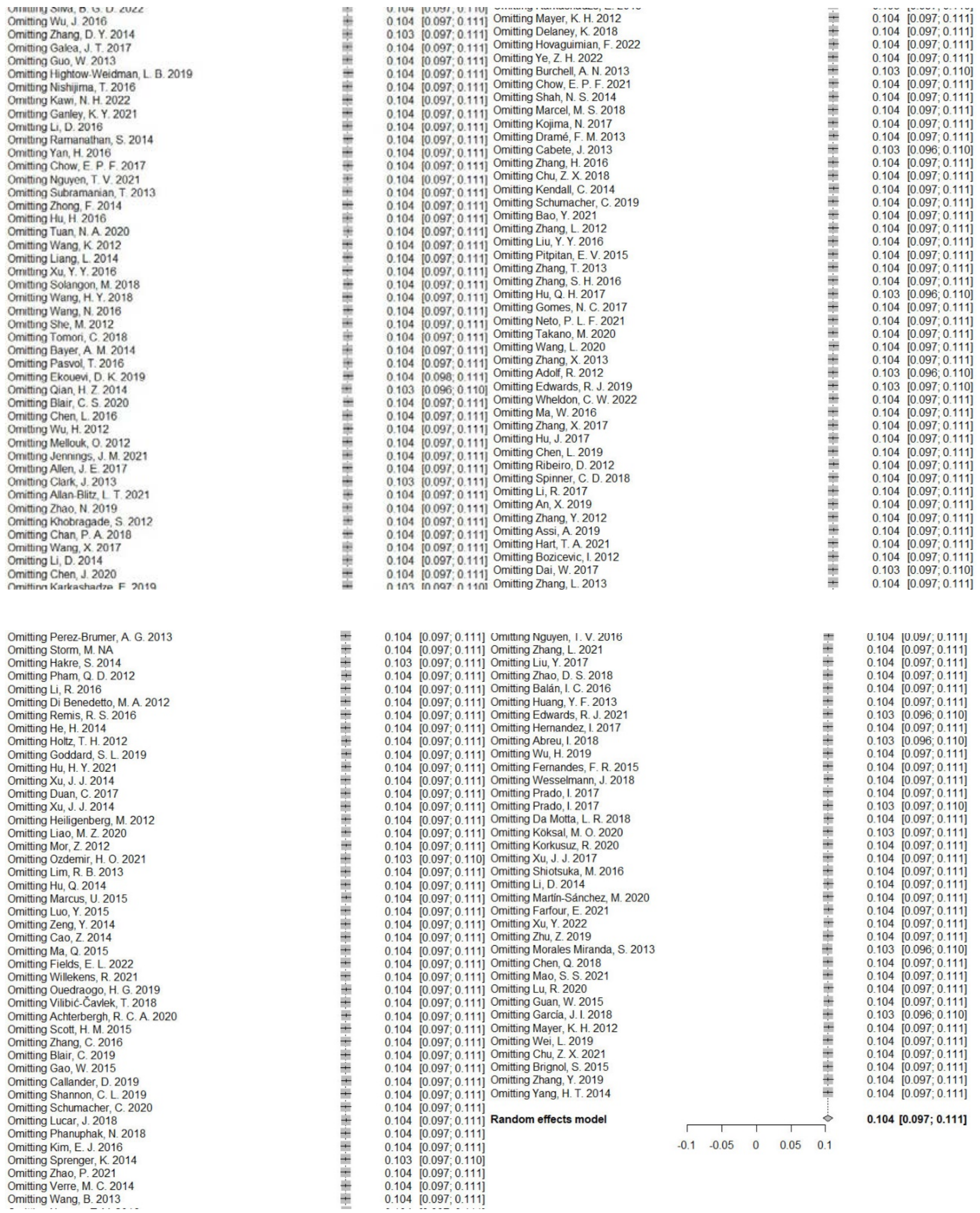
Omitting Silva, B. G. D. 2022 0.104 [0.097; 0.110]
Omitting Wu, J. 2016 0.104 [0.097; 0.111]
Omitting Zhang, D. Y. 2014 0.103 [0.097; 0.111]
Omitting Galea, J. T. 2017 0.104 [0.097; 0.111]
Omitting Guo, W. 2013 0.104 [0.097; 0.111]
Omitting Hightow-Weidman, L. B. 2019 0.104 [0.097; 0.111]
Omitting Nishijima, T. 2016 0.104 [0.097; 0.111]
Omitting Kawi, N. H. 2022 0.104 [0.097; 0.111]
Omitting Ganley, K. Y. 2021 0.104 [0.097; 0.111]
Omitting Li, D. 2016 0.104 [0.097; 0.111]
Omitting Ramanathan, S. 2014 0.104 [0.097; 0.111]
Omitting Yan, H. 2016 0.104 [0.097; 0.111]
Omitting Chow, E. P. F. 2017 0.104 [0.097; 0.111]
Omitting Nguyen, T. V. 2021 0.104 [0.097; 0.111]
Omitting Subramanian, T. 2013 0.104 [0.097; 0.111]
Omitting Zhong, F. 2014 0.104 [0.097; 0.111]
Omitting Hu, H. 2016 0.104 [0.097; 0.111]
Omitting Tuan, N. A. 2020 0.104 [0.097; 0.111]
Omitting Wang, K. 2012 0.104 [0.097; 0.111]
Omitting Liang, L. 2014 0.104 [0.097; 0.111]
Omitting Xu, Y. Y. 2016 0.104 [0.097; 0.111]
Omitting Solangon, M. 2018 0.104 [0.097; 0.111]
Omitting Wang, H. Y. 2018 0.104 [0.097; 0.111]
Omitting Wang, N. 2016 0.104 [0.097; 0.111]
Omitting She, M. 2012 0.104 [0.097; 0.111]
Omitting Tomori, C. 2018 0.104 [0.097; 0.111]
Omitting Bayer, A. M. 2014 0.104 [0.097; 0.111]
Omitting Pasvol, T. 2016 0.104 [0.097; 0.111]
Omitting Ekouevi, D. K. 2019 0.104 [0.098; 0.111]
Omitting Qian, H. Z. 2014 0.103 [0.096; 0.110]
Omitting Blair, C. S. 2020 0.104 [0.097; 0.111]
Omitting Chen, L. 2016 0.104 [0.097; 0.111]
Omitting Wu, H. 2012 0.104 [0.097; 0.111]
Omitting Mellouk, O. 2012 0.104 [0.097; 0.111]
Omitting Jennings, J. M. 2021 0.104 [0.097; 0.111]
Omitting Allen, J. E. 2017 0.104 [0.097; 0.111]
Omitting Clark, J. 2013 0.103 [0.097; 0.111]
Omitting Allan-Blitz, L. T. 2021 0.104 [0.097; 0.111]
Omitting Zhao, N. 2019 0.104 [0.097; 0.111]
Omitting Khobragade, S. 2012 0.104 [0.097; 0.111]
Omitting Chan, P. A. 2018 0.104 [0.097; 0.111]
Omitting Wang, X. 2017 0.104 [0.097; 0.111]
Omitting Li, D. 2014 0.104 [0.097; 0.111]
Omitting Chen, J. 2020 0.104 [0.097; 0.111]
Omitting Karkashadze, E. 2019 0.103 [0.097; 0.110]
Omitting Mayer, K. H. 2012 0.104 [0.097; 0.111]
Omitting Delaney, K. 2018 0.104 [0.097; 0.111]
Omitting Hovaguimian, F. 2022 0.104 [0.097; 0.111]
Omitting Ye, Z. H. 2022 0.104 [0.097; 0.111]
Omitting Burchell, A. N. 2013 0.103 [0.097; 0.110]
Omitting Chow, E. P. F. 2021 0.104 [0.097; 0.111]
Omitting Shah, N. S. 2014 0.104 [0.097; 0.111]
Omitting Marcel, M. S. 2018 0.104 [0.097; 0.111]
Omitting Kojima, N. 2017 0.104 [0.097; 0.111]
Omitting Dramé, F. M. 2013 0.104 [0.097; 0.111]
Omitting Cabete, J. 2013 0.103 [0.096; 0.110]
Omitting Zhang, H. 2016 0.104 [0.097; 0.111]
Omitting Chu, Z. X. 2018 0.104 [0.097; 0.111]
Omitting Kendall, C. 2014 0.104 [0.097; 0.111]
Omitting Schumacher, C. 2019 0.104 [0.097; 0.111]
Omitting Bao, Y. 2021 0.104 [0.097; 0.111]
Omitting Zhang, L. 2012 0.104 [0.097; 0.111]
Omitting Liu, Y. Y. 2016 0.104 [0.097; 0.111]
Omitting Pitpitan, E. V. 2015 0.104 [0.097; 0.111]
Omitting Zhang, T. 2013 0.104 [0.097; 0.111]
Omitting Zhang, S. H. 2016 0.104 [0.097; 0.111]
Omitting Hu, Q. H. 2017 0.103 [0.096; 0.110]
Omitting Gomes, N. C. 2017 0.104 [0.097; 0.111]
Omitting Neto, P. L. F. 2021 0.104 [0.097; 0.111]
Omitting Takano, M. 2020 0.104 [0.097; 0.111]
Omitting Wang, L. 2020 0.104 [0.097; 0.111]
Omitting Zhang, X. 2013 0.104 [0.097; 0.111]
Omitting Adolf, R. 2012 0.103 [0.096; 0.110]
Omitting Edwards, R. J. 2019 0.103 [0.097; 0.110]
Omitting Wheldon, C. W. 2022 0.104 [0.097; 0.111]
Omitting Ma, W. 2016 0.104 [0.097; 0.111]
Omitting Zhang, X. 2017 0.104 [0.097; 0.111]
Omitting Hu, J. 2017 0.104 [0.097; 0.111]
Omitting Chen, L. 2019 0.104 [0.097; 0.111]
Omitting Ribeiro, D. 2012 0.104 [0.097; 0.111]
Omitting Spinner, C. D. 2018 0.104 [0.097; 0.111]
Omitting Li, R. 2017 0.104 [0.097; 0.111]
Omitting An, X. 2019 0.104 [0.097; 0.111]
Omitting Zhang, Y. 2012 0.104 [0.097; 0.111]
Omitting Assi, A. 2019 0.104 [0.097; 0.111]
Omitting Hart, T. A. 2021 0.104 [0.097; 0.111]
Omitting Bozicevic, I. 2012 0.104 [0.097; 0.111]
Omitting Dai, W. 2017 0.103 [0.097; 0.110]
Omitting Zhang, L. 2013 0.104 [0.097; 0.111]
Omitting Perez-Brumer, A. G. 2013 0.104 [0.097; 0.111]
Omitting Storm, M. NA 0.104 [0.097; 0.111]
Omitting Hakre, S. 2014 0.103 [0.097; 0.111]
Omitting Pham, Q. D. 2012 0.104 [0.097; 0.111]
Omitting Li, R. 2016 0.104 [0.097; 0.111]
Omitting Di Benedetto, M. A. 2012 0.104 [0.097; 0.111]
Omitting Remis, R. S. 2016 0.104 [0.097; 0.111]
Omitting He, H. 2014 0.104 [0.097; 0.111]
Omitting Holtz, T. H. 2012 0.104 [0.097; 0.111]
Omitting Goddard, S. L. 2019 0.104 [0.097; 0.111]
Omitting Hu, H. Y. 2021 0.104 [0.097; 0.111]
Omitting Xu, J. J. 2014 0.104 [0.097; 0.111]
Omitting Duan, C. 2017 0.104 [0.097; 0.111]
Omitting Xu, J. J. 2014 0.104 [0.097; 0.111]
Omitting Heiligenberg, M. 2012 0.104 [0.097; 0.111]
Omitting Liao, M. Z. 2020 0.104 [0.097; 0.111]
Omitting Mor, Z. 2012 0.104 [0.097; 0.111]
Omitting Ozdemir, H. O. 2021 0.103 [0.097; 0.110]
Omitting Lim, R. B. 2013 0.104 [0.097; 0.111]
Omitting Hu, Q. 2014 0.104 [0.097; 0.111]
Omitting Marcus, U. 2015 0.104 [0.097; 0.111]
Omitting Luo, Y. 2015 0.104 [0.097; 0.111]
Omitting Zeng, Y. 2014 0.104 [0.097; 0.111]
Omitting Cao, Z. 2014 0.104 [0.097; 0.111]
Omitting Ma, Q. 2015 0.104 [0.097; 0.111]
Omitting Fields, E. L. 2022 0.104 [0.097; 0.111]
Omitting Willekens, R. 2021 0.104 [0.097; 0.111]
Omitting Ouedraogo, H. G. 2019 0.104 [0.097; 0.111]
Omitting Vilibić-Čavlek, T. 2018 0.104 [0.097; 0.111]
Omitting Achterbergh, R. C. A. 2020 0.104 [0.097; 0.111]
Omitting Scott, H. M. 2015 0.104 [0.097; 0.111]
Omitting Zhang, C. 2016 0.104 [0.097; 0.111]
Omitting Blair, C. 2019 0.104 [0.097; 0.111]
Omitting Gao, W. 2015 0.104 [0.097; 0.111]
Omitting Callander, D. 2019 0.104 [0.097; 0.111]
Omitting Shannon, C. L. 2019 0.104 [0.097; 0.111]
Omitting Schumacher, C. 2020 0.104 [0.097; 0.111]
Omitting Lucar, J. 2018 0.104 [0.097; 0.111]
Omitting Phanuphak, N. 2018 0.104 [0.097; 0.111]
Omitting Kim, E. J. 2016 0.104 [0.097; 0.111]
Omitting Sprenger, K. 2014 0.103 [0.097; 0.110]
Omitting Zhao, P. 2021 0.104 [0.097; 0.111]
Omitting Verre, M. C. 2014 0.104 [0.097; 0.111]
Omitting Wang, B. 2013 0.104 [0.097; 0.111]
Omitting Nguyen, T. V. 2016 0.104 [0.097; 0.111]
Omitting Zhang, L. 2021 0.104 [0.097; 0.111]
Omitting Liu, Y. 2017 0.104 [0.097; 0.111]
Omitting Zhao, D. S. 2018 0.104 [0.097; 0.111]
Omitting Balán, I. C. 2016 0.104 [0.097; 0.111]
Omitting Huang, Y. F. 2013 0.104 [0.097; 0.111]
Omitting Edwards, R. J. 2021 0.103 [0.096; 0.110]
Omitting Hernandez, I. 2017 0.104 [0.097; 0.111]
Omitting Abreu, I. 2018 0.103 [0.096; 0.110]
Omitting Wu, H. 2019 0.104 [0.097; 0.111]
Omitting Fernandes, F. R. 2015 0.104 [0.097; 0.111]
Omitting Wesselmann, J. 2018 0.104 [0.097; 0.111]
Omitting Prado, I. 2017 0.104 [0.097; 0.111]
Omitting Prado, I. 2017 0.103 [0.097; 0.110]
Omitting Da Motta, L. R. 2018 0.104 [0.097; 0.111]
Omitting Köksal, M. O. 2020 0.103 [0.097; 0.111]
Omitting Korkusuz, R. 2020 0.104 [0.097; 0.111]
Omitting Xu, J. J. 2017 0.104 [0.097; 0.111]
Omitting Shiotsuka, M. 2016 0.104 [0.097; 0.111]
Omitting Li, D. 2014 0.104 [0.097; 0.111]
Omitting Martín-Sánchez, M. 2020 0.104 [0.097; 0.111]
Omitting Farfour, E. 2021 0.104 [0.097; 0.111]
Omitting Xu, Y. 2022 0.104 [0.097; 0.111]
Omitting Zhu, Z. 2019 0.104 [0.097; 0.111]
Omitting Morales Miranda, S. 2013 0.103 [0.096; 0.110]
Omitting Chen, Q. 2018 0.104 [0.097; 0.111]
Omitting Mao, S. S. 2021 0.104 [0.097; 0.111]
Omitting Lu, R. 2020 0.104 [0.097; 0.111]
Omitting Guan, W. 2015 0.104 [0.097; 0.111]
Omitting García, J. I. 2018 0.103 [0.096; 0.110]
Omitting Mayer, K. H. 2012 0.104 [0.097; 0.111]
Omitting Wei, L. 2019 0.104 [0.097; 0.111]
Omitting Chu, Z. X. 2021 0.104 [0.097; 0.111]
Omitting Brignol, S. 2015 0.104 [0.097; 0.111]
Omitting Zhang, Y. 2019 0.104 [0.097; 0.111]
Omitting Yang, H. T. 2014 0.104 [0.097; 0.111]
Random effects model 0.104 [0.097; 0.111]
-0.1 -0.05 0 0.05 0.1

Figure S2. Sensitivity analysis of syphilis incidence study

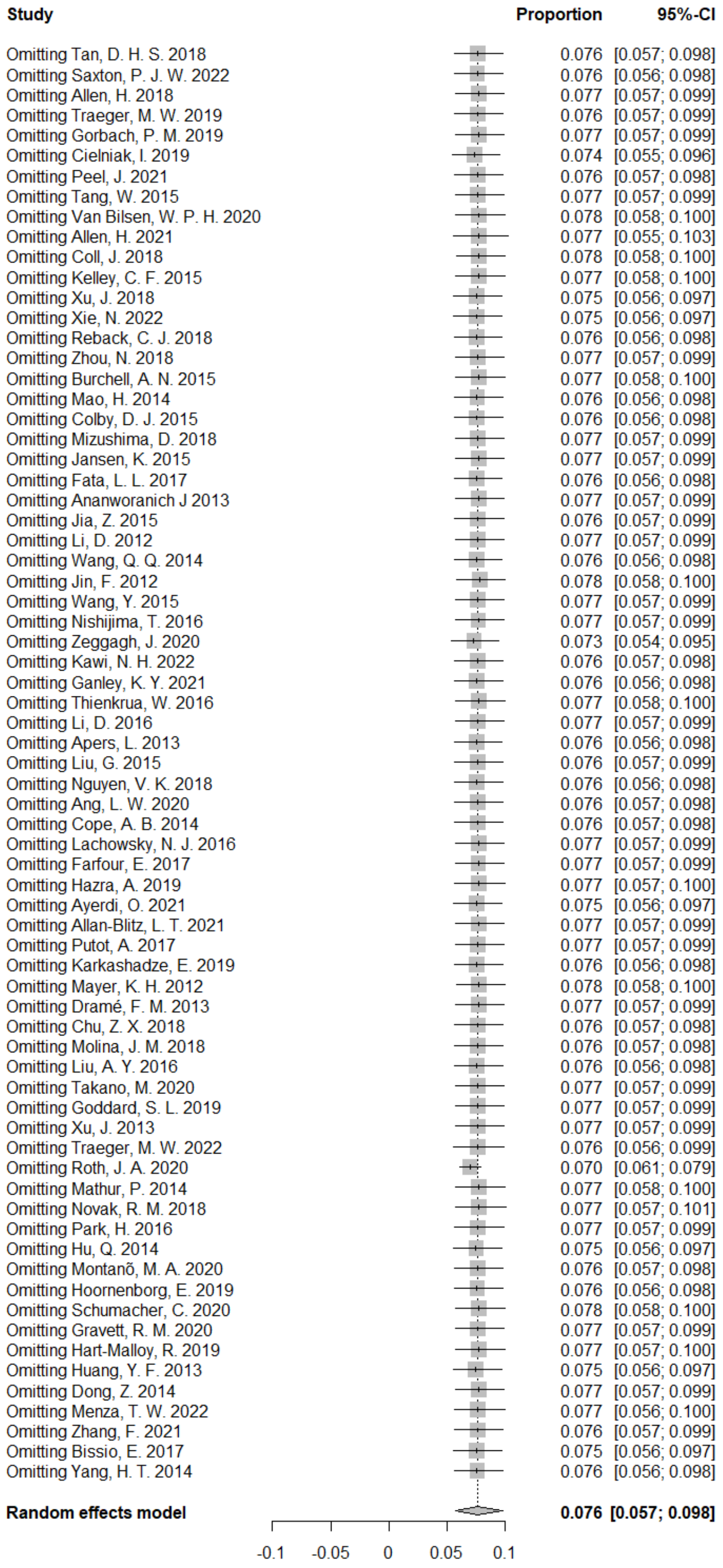

Figure S3. Funnel plot and publication bias

Pooled estimates of syphilis prevalence

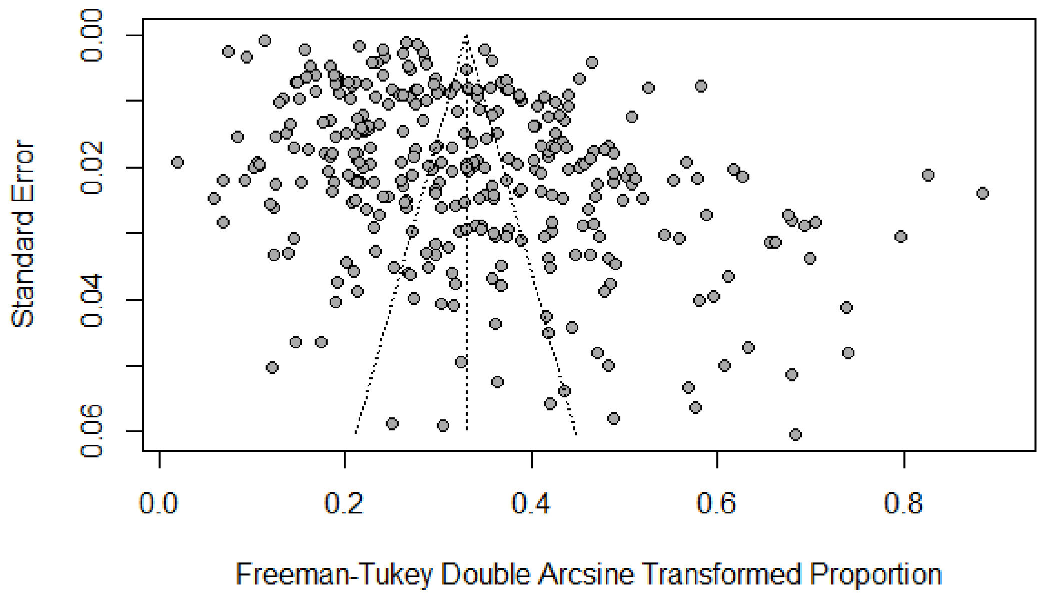

Egger test: p<0.05

After Trim-and-fill:

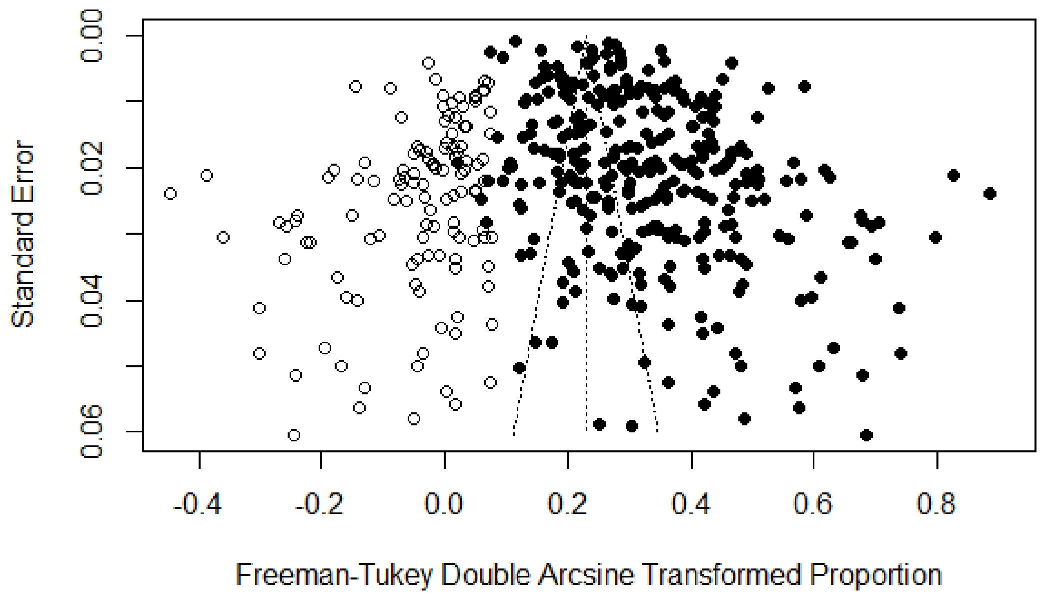

K=464 (with 126 added studies)   5.05%   4.56; 5.56%

Pooled estimates of syphilis incidence

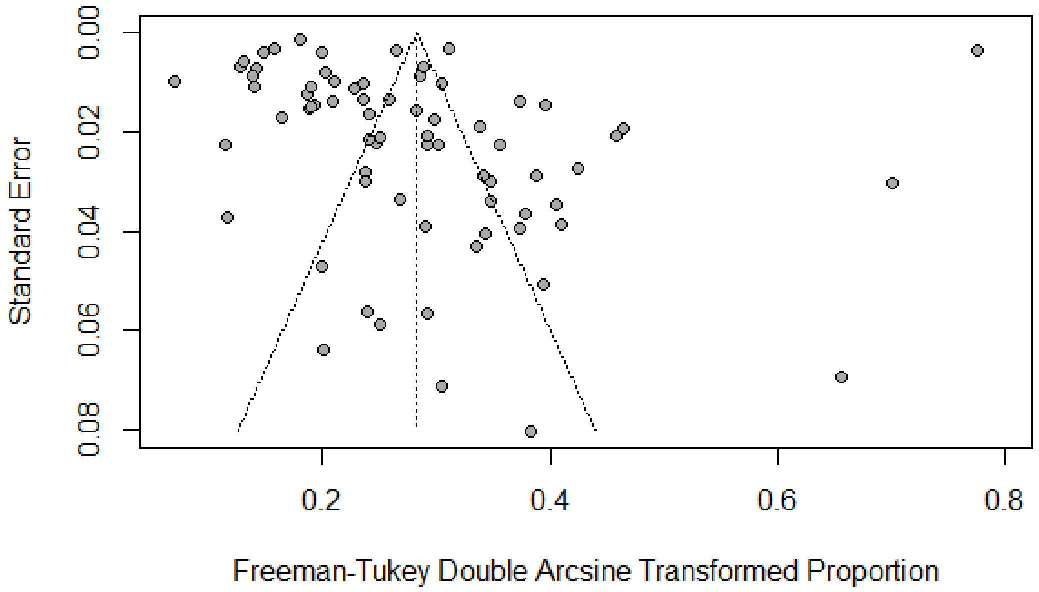

Egger test: p=0.365

Figure S4. Estimated syphilis incidence in MSM by country

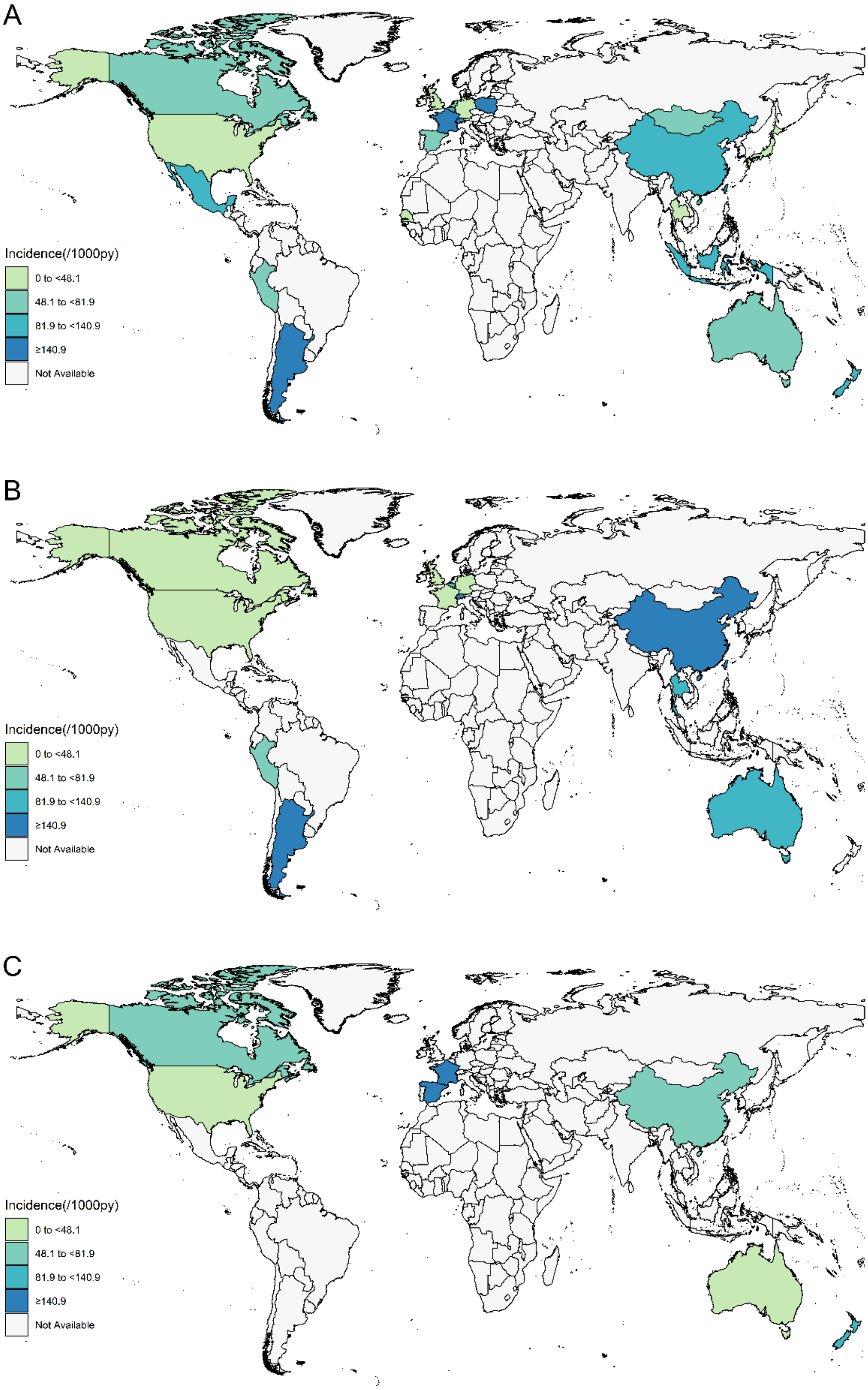

(A) Syphilis incidence in overall MSM population; (B) Syphilis incidence in HIV-positive MSM subpopulation; (C) Syphilis incidence in HIV-negative MSM subpopulation;

Figure S5. Associations (odds ratios) between demographic, social behavior factors and syphilis infection in different geographic regions

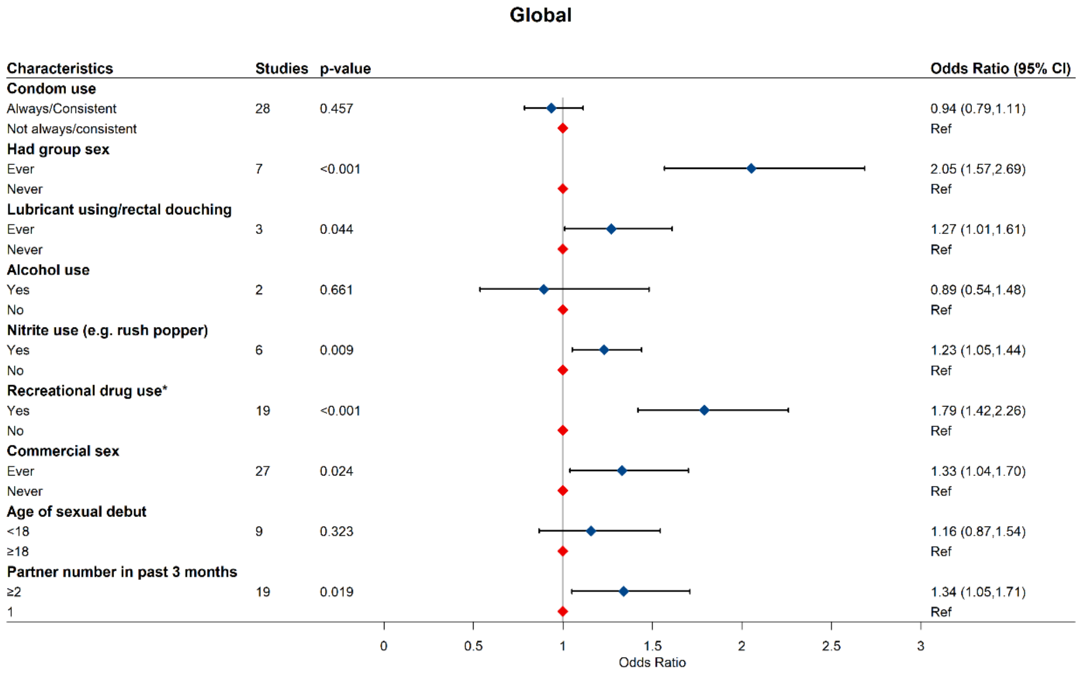

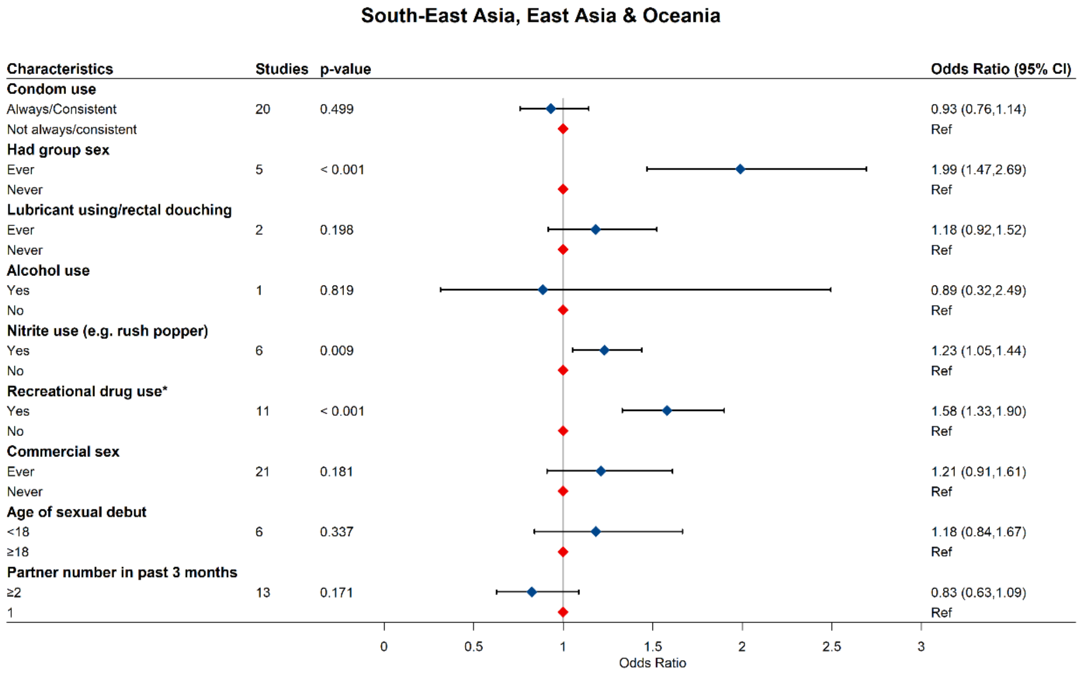

South Asia

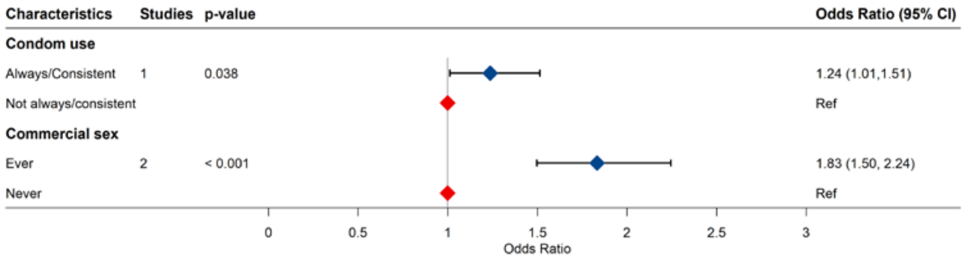

North Africa & Middle East

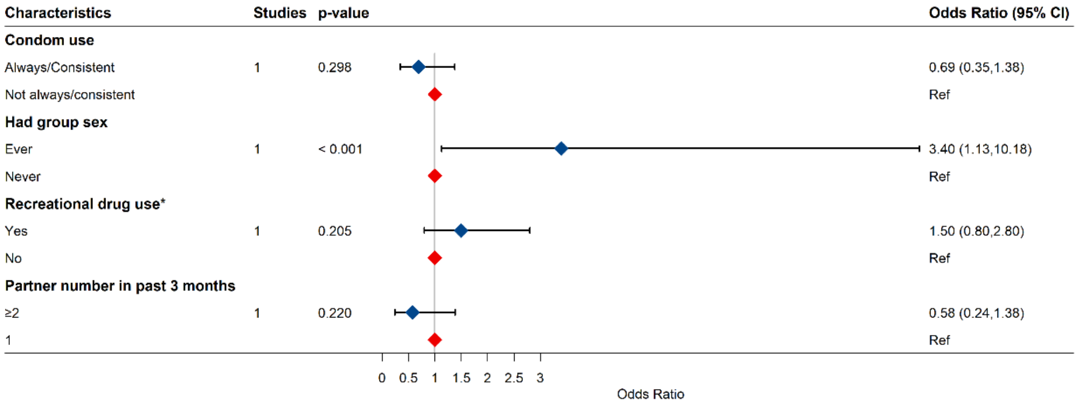

High Income

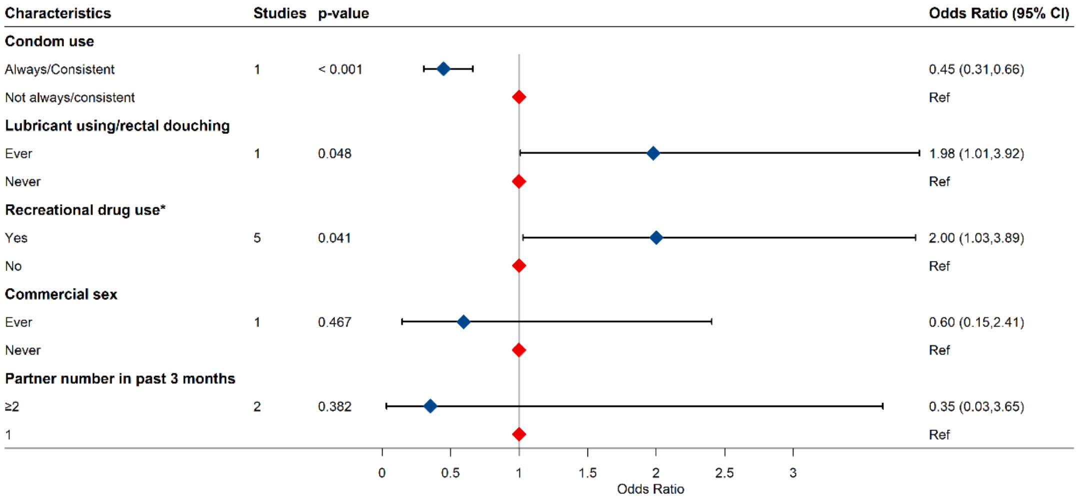

Latin America & Caribbean

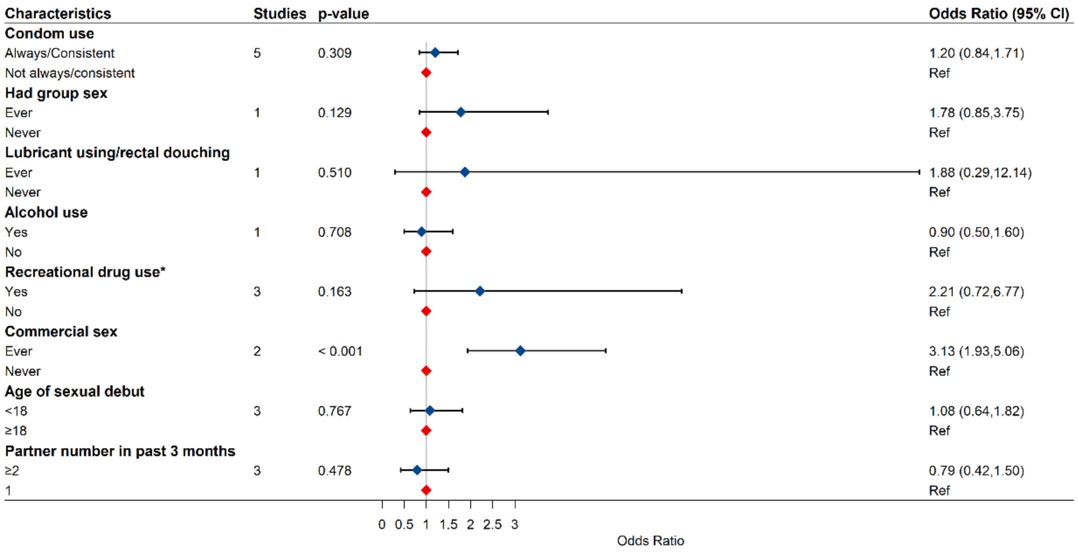

Central Europe, Eastern Europe & Central Asia

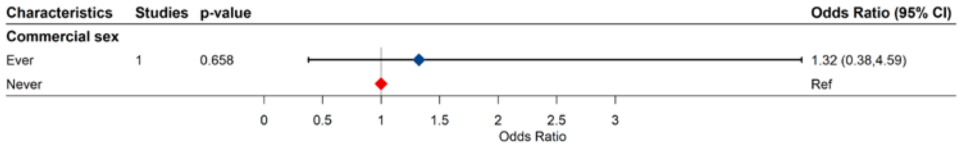

Table S1. Characteristics of the included prevalence studies.

**Author, Year**	**Country**	**Region**	**Sample size**	**Testing**	**Study design**	**Sampling methods**	**Study Quality**	**Selection score**	**Comparability score**	**Outcome score**
Abreu, I.,2018	Portugal	High Income	94	VDRL+TPPA	Cross-Sectional	Convenient	8	3	2	3
Achterbergh, R. C. A.,2020	Netherlands	High Income	4455	not mentioned	Cross-Sectional	Convenient	6	3	0	3
Adolf, R.,2012	Brazil	Latin America & Caribbean	317	VDRL+FTA	Cohort(Baseline)	Convenient	7	3	1	3
Allan-Blitz, L. T.,2021	US	High Income	1518	RPR+TPPA	Cohort(Baseline)	Convenient	8	3	2	3
Allen, J. E.,2017	US	High Income	853	self-reported	Cross-Sectional	Convenient	6	3	1	2
An, X.,2019	China	South-East Asia, East Asia & Oceania	267	RPR+TPPA	Cross-Sectional	Convenient	7	3	1	3
Ananworanich J,2013	Thailand	South-East Asia, East Asia & Oceania	4324	RPR+immunochromatography	Cohort(Baseline)	Convenient	8	4	1	3
Assi, A.,2019	Lebanon	North Africa & Middle East	1429	rapid test+VDRL	Cross-Sectional	Convenient	6	3	0	3
Azyei, I.,2014	Mongolia	Central Europe, Eastern Europe & Central Asia	196	RPR+TPHA	Cross-Sectional	RDS	7	4	1	2
Bai, J.,2019	China	South-East Asia, East Asia & Oceania	1200	TP-ELISA+TRUST	Cross-Sectional	Senital Survelliance	7	4	1	2
Bai, J.,2022	China	South-East Asia, East Asia & Oceania	3616	TP-ELISA+TRUST	Cross-Sectional	Convenient	7	4	1	2
Balán, I. C.,2016	Argentina	High Income	496	not mentioned	Cross-Sectional	RDS	4	2	0	2
Balán, I. C.,2018	Argentina	High Income	226	VDRL+TPHA	Cross-Sectional	RDS	6	3	1	2
Bao, Y.,2021	Australia	High Income	13820	TP serology	Cross-Sectional	Convenient	6	3	0	3
Bayer, A. M.,2014	Peru	Latin America & Caribbean	80	RPR+TPPA	Cross-Sectional	Purposive	9	4	2	3

Beymer, M. R.,2016	US	High Income	2735	RPR	Cohort(Baseline)	Convenient Sampling	7	3	1	3
Blair, C. S.,2020	US	High Income	1140	RPR+TPPA	Cross-Sectional	convenient	7	4	1	2
Blair, C.,2019	Peru	Latin America & Caribbean	898	RPR+TPHA	Cross-Sectional	Convenient	6	3	0	3
Bozicevic, I.,2012	Croatia	Central Europe, Eastern Europe & Central Asia	387	TPHA+RPR	Cross-Sectional	RDS	6	3	0	3
Brignol, S.,2015	Brazil	Latin America & Caribbean	363	rapid antibody test	Cross-Sectional	RDS	6	3	0	3
Brignol, S.,2016	Brazil	Latin America & Caribbean	371	TP antibody	Cross-Sectional	RDS	7	4	1	2
Burchell, A. N.,2013	Canada	High Income	4036	RPR+FTA	Cohort(Baseline)	Convenient	6	3	0	3
Cabete, J.,2013	Portugal	High Income	108	not mentioned	Cross-Sectional	Convenient	5	2	0	3
Cai, R.,2014	China	South-East Asia, East Asia & Oceania	649	RPR+TPPA	Cross-Sectional	RDS	7	4	1	2
Cai, Y. M.,2017	China	South-East Asia, East Asia & Oceania	3109	TRUST+TPPA	Cross-Sectional	Snow-Ball+RDS	8	4	1	3
Callander, D.,2017	Australia	High Income	22805	medical record	Cross-Sectional	Convenient Sampling	6	3	1	2
Callander, D.,2019	Australia	High Income	48347	not mentioned	Cross-Sectional	Sentinel Surveillance	8	3	2	3
Cao, Z.,2014	China	South-East Asia, East Asia & Oceania	858	RPR+TPPA	Cross-Sectional	Snowball	6	3	0	3
Chaiyabutr, C.,2022	Thailand	South-East Asia, East Asia & Oceania	270	serology	Cross-Sectional	Convenient Sampling	6	3	0	3
Chan, P. A.,2018	US	High Income	415	clinical outcome	Cross-Sectional	Convenient	8	3	2	3
Chang, Y. H.,2014	Taiwan	South-East Asia, East Asia & Oceania	299	RPR+TPPA	Cross-Sectional	Convenient Sampling	7	3	1	3

Chen, J. S.,2022	Vietnam	South-East Asia, East Asia & Oceania	205	RPR+TPHA	Cross-Sectional	Convenient	7	4	1	2
Chen, J.,2020	China	South-East Asia, East Asia & Oceania	1151	TRUST+TPPA	Cross-Sectional	Convenient	8	3	2	3
Chen, L.,2016	China	South-East Asia, East Asia & Oceania	2958	RPR+TPPA	Cross-Sectional	RDS And Snow-Ball	7	3	1	3
Chen, L.,2019	China	South-East Asia, East Asia & Oceania	3616	RPR+TPPA	Cross-Sectional	Convenient	5	2	1	2
Chen, Q.,2018	China	South-East Asia, East Asia & Oceania	5283	not mentioned	Cross-Sectional	Snowball	8	3	2	3
Chen, X.,2015	China	South-East Asia, East Asia & Oceania	826	PRP+TPPA	Cross-Sectional	Venue-Based, Peer Referral Using "Snowball" Techniques, And Internet Advertisements	8	4	1	3
Chen, Y.,2017	China	South-East Asia, East Asia & Oceania	19750	ELISA+TRUST	Cross-Sectional	Snowball Sampling, Venue-Based Sampling, And Online Recruitment	7	3	1	3
Chow, E. P. F.,2017	Australia	High Income	84547	RPR+TP immunoassays	Cross-Sectional	National Survelliance	8	4	1	3
Chow, E. P. F.,2021	Australia	High Income	357	serology	Cross-Sectional	Convenient	7	3	1	3
Chow, J. Y.,2017	Peru	Latin America & Caribbean	312	RPR+TPPA	Cohort(Baseline)	Convenient Sampling	7	3	1	3
Chu, Z. X.,2018	China	South-East Asia, East Asia & Oceania	657	RPR+TPPA	Cohort(Baseline)	Snowball	6	3	0	3
Chu, Z. X.,2021	China	South-East Asia, East Asia & Oceania	515	RPR+TPPA	Cross-Sectional	Convenient	5	3	0	2

Chua, A. C.,2013	Singapore	High Income	72	not mentioned	Cross-Sectional	RDS	5	2	1	2
Clark, J.,2013	Peru	Latin America & Caribbean	532	RPR+TPPA	Cross-Sectional	Convenient	7	3	1	3
Colby, D. J.,2015	Thailand	South-East Asia, East Asia & Oceania	188	VDRL+TPHA	Cohort(Baseline)	Convenient	6	3	1	2
Coll, J.,2018	Spain	High Income	250	nontrponemal+treponemal	Cohort(Baseline)	Convenient	6	3	1	2
Colón-López, V.,2013	Puerto Rico	Latin America & Caribbean	78	RPR	Cross-Sectional	Convenient	6	3	1	2
Courjon, J.,2015	France	High Income	176	TPHA+specific T. pallidum nested polymerase chain reaction (nPCR)	Cross-Sectional	Convenient Sampling	5	2	1	2
Creswell, J.,2012	El Salvador	Latin America & Caribbean	791	RPR+TPHA	Cross-Sectional	RDS	7	4	1	2
Cunha, C. B.,2015	Brazil	Latin America & Caribbean	284	RPR+TPHA	Cross-Sectional	Convenient Sampling	8	4	1	3
Da Motta, L. R.,2018	Brazil	Latin America & Caribbean	1279	VDRL+TP antibody	Cross-Sectional	Convenient	5	2	1	2
Dai, W.,2017	China	South-East Asia, East Asia & Oceania	533	ELISA+TRUST	Cross-Sectional	Convenient	8	3	2	3
Das, A.,2015	China	South-East Asia, East Asia & Oceania	2936	RPR and TPPA	Cross-Sectional	Snow-Ball	9	4	2	3
Delaney, K.,2018	US	High Income	501	not mentioned	Cross-Sectional	Convenient	7	3	1	3
Di Benedetto, M. A.,2012	Italy	High Income	74	RPR+TP antibody	Cross-Sectional	Convenient	5	2	0	3
Di Tullio, F.,2022	Italy	High Income	186	RPR+TPPA	Cohort(Baseline)	Convenient	6	2	1	3
Dorjiwangmo,2017	China	South-East Asia, East Asia & Oceania	102	TRUST+TP antibody	Cross-Sectional	Simple Random	7	4	1	2
Dramé, F.	Senegal	Sub-saharan Africa	115	RPR+TP antibody	Cohort(Baseline)	Convenient	5	1	2	2

M.,2013										
Duan, C.,2017	China	South-East Asia, East Asia & Oceania	1935	RPR+TPPA	Cross-Sectional	Time-Location, RDS , Snow-Ball	7	3	1	3
Edwards, R. J.,2019	Trinidad and Tobago	Latin America & Caribbean	68	VDRL+TPPA	Cross-Sectional	Convenient	6	2	1	3
Edwards, R. J.,2021	Trinidad and Tobago	Latin America & Caribbean	218	VDRL+TPPA	Cross-Sectional	Convenient	6	3	1	2
Edwards, R. J.,2022	Trinidad and Tobago	Latin America & Caribbean	264	VDRL+TPPA	Cross-Sectional	Convenient Sampling	7	3	1	3
Ekouevi, D. K.,2019	Togo	Sub-saharan Africa	678	Syphilis rapid test	Cross-Sectional	RDS	6	3	0	3
Fan, S.,2012	China	South-East Asia, East Asia & Oceania	501	RPR+TPPA	Cross-Sectional	RDS	7	3	1	3
Fan, W.,2014	China	South-East Asia, East Asia & Oceania	887	RPR+TPPA	Cross-Sectional	Convenient	7	3	1	3
Fan, W.,2016	China	South-East Asia, East Asia & Oceania	391	TPPA	Cross-Sectional	Convenient Sampling	7	4	1	2
Farfour, E.,2021	France	High Income	781	RPR or VDRL+TPHA	Cross-Sectional	Convenient	7	3	1	3
Fata, L. L.,2017	France	High Income	211	serology	Cohort(Baseline)	Convenient	6	3	1	2
Fernandes, F. R.,2015	Brazil	Latin America & Caribbean	278	VDRL+TP antibody	Cross-Sectional	Convenient	8	3	2	3
Fields, E. L.,2022	US	High Income	151	not mentioned	Cross-Sectional	Convenient	5	2	0	3
Figueroa, J. P.,2013	Jamaica	Latin America & Caribbean	201	TRUST+TPPA	Cross-Sectional	Convenient	7	4	1	2
Fu, G. F.,2015	China	South-East Asia, East Asia & Oceania	413	TRUST+ELISA	Cross-Sectional	Convenient	7	4	1	2

Fuchs, W.,2016	Germany	High Income	503	RPR+TPLA	Cross-Sectional	Convenient Sampling	6	3	0	3
Galárraga, O.,2014	Mexico	Latin America & Caribbean	267	TP antibody+VDRL	Cohort(Baseline)	Convenient Sampling	6	2	1	3
Galea, J. T.,2017	Peru	Latin America & Caribbean	283	rapid TP test	Cross-Sectional	Snow-Ball	7	4	1	2
Ganley, K. Y.,2021	Mexico	Latin America & Caribbean	227	VDRL+TP antibody	Cohort(Baseline)	Convenient	8	4	1	3
Gao, W.,2015	China	South-East Asia, East Asia & Oceania	600	RPR+TPPA	Cross-Sectional	Snowball	6	3	0	3
García, J. I.,2018	Guatemala	Latin America & Caribbean	338	VDRL+TPHA	Cross-Sectional	Convenient	6	3	0	3
Gianella, S.,2015	US	High Income	131	RPR and TPPA	Cohort(Baseline)	Convenient	5	2	1	2
Goddard, S. L.,2019	Australia	High Income	617	EIA+TPPA/FTA+RPR	Cohort(Baseline)	Convenient	6	2	1	3
Gomes, N. C.,2017	Brazil	Latin America & Caribbean	702	rapid test+VDRL	Cross-Sectional	Convenient	8	4	1	3
Goodman, S. H.,2016	Burkina Faso	Sub-saharan Africa	672	RPR+rapid test	Cross-Sectional	RDS	8	4	1	3
Grewal, R.,2019	Canada	High Income	2632	CMIA+RPR+TPPA/FTA-ABS	Cohort(Baseline)	Convenient Sampling	8	3	2	3
Guan, W.,2015	China	South-East Asia, East Asia & Oceania	6645	RPR+TPPA	Cross-Sectional	Convenient	6	3	0	3
Guanghua, L.,2018	China	South-East Asia, East Asia & Oceania	5658	RPR +ELISA	Cross-Sectional	Convenient	7	4	1	2
Gulov, K.,2016	Tajikistan	Central Europe, Eastern Europe & Central Asia	502	TP antibody	Cross-Sectional	Convenient	8	4	1	3
Guo, W.,2013	China	South-East Asia, East Asia & Oceania	167	RPR+TPHA	Cohort(Baseline)	Convenient	6	2	1	3

Guo, Y. L.,2013	China	South-East Asia, East Asia & Oceania	655	ELISA+TURST	Cross-Sectional	Snow-Ball	7	3	1	3
Guo, Y.,2012	China	South-East Asia, East Asia & Oceania	307	serology	Cross-Sectional	Convenient Sampling	7	3	1	3
Guo, Y.,2014	China	South-East Asia, East Asia & Oceania	657	TRUST+TP antibody	Cross-Sectional	Snow-Ball+Convenient	7	3	1	3
Hakre, S.,2014	Panama	Latin America & Caribbean	600	RPR+TPHA	Cross-Sectional	RDS	7	3	1	3
Hall, C. D. X.,2020	China	South-East Asia, East Asia & Oceania	546	TPPA	Cross-Sectional	Convenient Sampling	7	3	1	3
Ham, C.,2014	El Salvador	Latin America & Caribbean	710	RPR/TPPA	Cross-Sectional	RDS	5	1	1	3
Ham, D.,2022	El Salvador	Latin America & Caribbean	620	RPR+TPPA	Cross-Sectional	RDS	6	4	1	1
Ham, D.,2022	Guatemala	Latin America & Caribbean	486	RPR+TPPA	Cross-Sectional	RDS	6	4	1	1
Ham, D.,2022	Honduras	Latin America & Caribbean	553	RPR+TPPA	Cross-Sectional	RDS	6	4	1	1
Ham, D.,2022	Nicaragua	Latin America & Caribbean	590	RPR+TPPA	Cross-Sectional	RDS	6	4	1	1
Hao, C.,2014	China	South-East Asia, East Asia & Oceania	220	RPR+TPPA	Cross-Sectional	Convenient Sampling	6	3	1	2
Hart, T. A.,2021	Canada	High Income	2449	RPR+TPPA	Cross-Sectional	RDS	7	3	1	3
He, H.,2014	China	South-East Asia, East Asia & Oceania	200	RPR+TPPA	Cross-Sectional	Snow-Ball	7	2	2	3
Heiligenberg, M.,2012	Netherlands	High Income	658	TPHA+RPR/VDRL	Cross-Sectional	Convenient	7	4	1	2
Heiligenberg, M.,2012	Netherlands	High Income	673	RPR/VDRL/FTA+TPPA	Cross-Sectional	Convenient	7	3	1	3
Hernandez,	Ecuador	Latin America &	291	VDRL+TPPA	Cross-Sectional	Convenient	8	3	2	3

I.,2017		Caribbean								
Hightow-Weidman, L. B.,2019	US	High Income	226	RPR+TP antibody	Cohort(Baseline)	Convenient	7	3	1	3
Hiransuthikul, A.,2018	Thailand	South-East Asia, East Asia & Oceania	1862	VDRL+TPHA	Cohort(Baseline)	Convenient	5	3	1	1
Holtz, T. H.,2012	Thailand	South-East Asia, East Asia & Oceania	1541	RPR+TP antibody	Cross-Sectional	Convenient	8	3	2	3
Hotton, A. L.,2012	US	High Income	1046	ELISA+FTA-ABS	Cross-Sectional	Convenient Sampling	7	3	1	3
Hovaguimian, F.,2022	Switzerland	High Income	728	RPR/VDRL+TPHA	Cohort(Baseline)	Probability	6	3	0	3
Hoyos-Mallecot, Y.,2022	Spain	High Income	6256	TP antibody +RPR	Cross-Sectional	Convenient Sampling	8	4	1	3
Hu, H. Y.,2021	China	South-East Asia, East Asia & Oceania	4469	nonspecific+specific antibody	Cross-Sectional	Convenient	8	3	2	3
Hu, H.,2016	China	South-East Asia, East Asia & Oceania	12853	TRUST+TP antibody	Cross-Sectional	Convenient	8	4	2	2
Hu, J.,2017	China	South-East Asia, East Asia & Oceania	15705	RPR+TP antibody	Cross-Sectional	Multistage Mixed Snow-Ball	8	3	2	3
Hu, Q. H.,2017	China	South-East Asia, East Asia & Oceania	545	RPR+TPPA	Cross-Sectional	Convenient	8	3	2	3
Hu, Q.,2014	China	South-East Asia, East Asia & Oceania	778	RPR+TPPA	Cohort(Baseline)	Convenient	9	4	2	3
Hu, Y.,2013	China	South-East Asia, East Asia & Oceania	671	RPR+TPPA	Cross-Sectional	Convenient Sampling+RDS	8	3	2	3
Huan, X.,2015	China	South-East Asia, East Asia & Oceania	407	RPR and TPPA	Cross-Sectional	RDS	9	4	2	3
Huang, D.,2014	China	South-East Asia, East Asia & Oceania	1239	RPR+TPHA	Cross-Sectional	RDS	7	4	1	2

Huang, P. X.,2020	China	South-East Asia, East Asia & Oceania	901	RPR+TPPA	Cross-Sectional	Convenient Sampling+RDS	6	3	0	3
Huang, Y. F.,2013	Taiwan	South-East Asia, East Asia & Oceania	361	RPR or VDRL+TPHA	Cohort(Baseline)	Convenient	4	2	0	2
Huang, Y.,2016	China	South-East Asia, East Asia & Oceania	965	RPR+TPPA	Cross-Sectional	Time-Location Sampling (Tls)	7	3	1	3
Hui, S.,2022	China	South-East Asia, East Asia & Oceania	2820	ELISA and TRUST	Cohort(Baseline)	Snow-Ball	7	4	1	2
Hung, P.,2020	Peru	Latin America & Caribbean	252	positive rapid treponemal tests+RPR	Cross-Sectional	Convenient	9	4	2	3
Jacobson, J. O.,2014	Ecuador	Latin America & Caribbean	414	RPR+FTA-ABS	Cross-Sectional	RDS	7	3	1	3
Jansen, K.,2015	Germany	High Income	1838	CLIA+TPPA+FTA-Abs/RPR	Cohort(Baseline)	Convenient	6	3	1	2
Javanbakht, M.,2020	US	High Income	445	serology	Cohort(Baseline)	Convenient Sampling	6	2	1	3
Jennings, J. M.,2021	US	High Income	268	RPR+TP antibody	Cross-Sectional	Convenient	7	3	1	3
Jia, Z.,2015	China	South-East Asia, East Asia & Oceania	3625	RPR+TPPA	Cohort(Baseline)	Convenient	7	3	1	3
Johnston, L. G.,2013	Dominican Republic	Latin America & Caribbean	1388	RPR+TP antibody	Cross-Sectional	RDS	7	4	1	2
Johnston, L. G.,2013	Morocco	North Africa & Middle East	669	VDRL+TPHA	Cross-Sectional	RDS	7	4	1	2
Jung, M.,2012	South Korea	High Income	108	TPPA	Cross-Sectional	Convenient Sampling	7	3	1	3
Karkashadze, E.,2019	US	High Income	154	not mentioned	Cohort(Baseline)	Convenient	7	3	1	3
Katz, D. A.,2018	US	High Income	230	RPR+TPPA	Cohort(Baseline)	Convenient	7	4	1	2

Kawi, N. H.,2022	Indonesia	South-East Asia, East Asia & Oceania	2912	RPR+rapid TP antibody	Cohort(Baseline)	Convenient	9	4	2	3
Kendall, C.,2014	Angola	Sub-saharan Africa	310	TP antibody	Cross-Sectional	RDS	6	3	0	3
Khobragade, S.,2012	India	South Asia	3229	serology	Cross-Sectional	Cluster	8	3	2	3
Kim, E. J.,2016	Uganda	Sub-saharan Africa	290	RPR+TP antibody	Cross-Sectional	RDS	8	3	2	3
Kojima, N.,2017	Peru	Latin America & Caribbean	312	RPR+TPPA	Cohort(Baseline)	Convenient	8	3	2	3
Köksal, M. O.,2020	Turkey	North Africa & Middle East	87	TP antibody	Cross-Sectional	Convenient	5	2	0	3
Korkusuz, R.,2020	Turkey	North Africa & Middle East	573	VDRL+TPHA	Cross-Sectional	Convenient	7	3	1	3
Kwan, T.,2018	Hongkong	South-East Asia, East Asia & Oceania	71	not mentioned	Cohort(Baseline)	Convenient Sampling	7	3	1	3
Lemmet, T.,2022	France	High Income	4277	VDRL/RPR+TPHA/ FTA/ELISA/CMIA	Cohort(Baseline)	Convenient	7	4	1	2
Li, D.,2012	China	South-East Asia, East Asia & Oceania	797	RPR+TPPA	Cohort(Baseline)	Convenient	7	3	2	2
Li, D.,2014	China	South-East Asia, East Asia & Oceania	400	TPPA	Cross-Sectional	Convenient	8	3	2	3
Li, D.,2014	China	South-East Asia, East Asia & Oceania	149848	TRUST+TP antibody	Cross-Sectional	Snowball+Convenient	7	3	1	3
Li, D.,2016	China	South-East Asia, East Asia & Oceania	962	RPR+TPPA	Cohort(Baseline)	Convenient	7	3	1	3
Li, Q.,2019	China	South-East Asia, East Asia & Oceania	523	RPR+TPPA	Cohort(Baseline)	Convenient	8	4	1	3
Li, R.,2016	China	South-East Asia, East Asia & Oceania	1316	not mentioned	Cross-Sectional	RDS	5	3	0	2
Li, R.,2017	China	South-East Asia, East Asia & Oceania	459	TRUST+TP antibody	Cross-Sectional	RDS	6	3	1	2

Liang, J.,2015	Hongkong	South-East Asia, East Asia & Oceania	452	serology	Cross-Sectional	Convenient Sampling	6	3	0	3
Liang, L.,2014	China	South-East Asia, East Asia & Oceania	2783	TRUST+TP antibody	Cross-Sectional	Convenient+Snow-Ball	9	4	2	3
Liao, M. Z.,2020	China	South-East Asia, East Asia & Oceania	1203	TRUST+ELISA	Cross-Sectional	Convenient	8	3	2	3
Liao, M.,2019	China	South-East Asia, East Asia & Oceania	3478	ELISA+TRUST	Cross-Sectional	Convenient Sampling+Snow-Ball Sampling	7	3	1	3
Lim, R. B.,2013	Singapore	High Income	127	RPR/VDRL+TPPA/TPHA/IgG	Cross-Sectional	Random	8	4	1	3
Lin, J. J.,2021	Taiwan	South-East Asia, East Asia & Oceania	1514	RPR titer>1:4	Cohort(Baseline)	Convenient	6	3	1	2
Liu, J.,2013	China	South-East Asia, East Asia & Oceania	388	Syphilis antibodies by ELISA	Cross-Sectional	Convenient	7	4	1	2
Liu, Y. Y.,2016	China	South-East Asia, East Asia & Oceania	587	TP antibody	Cross-Sectional	Convenient	6	3	0	3
Liu, Y.,2016	China	South-East Asia, East Asia & Oceania	3588	TRUST+TPPA	Cross-Sectional	Convenient Sampling+RDS	9	4	2	3
Liu, Y.,2016	China	South-East Asia, East Asia & Oceania	3588	Syphilis seropositivity	Cross-Sectional	Convenient Sampling+RDS	7	3	1	3
Liu, Y.,2017	China	South-East Asia, East Asia & Oceania	732	PRP+TPPA	Cross-Sectional	Convenient Sampling	7	3	1	3
Liu, Y.,2017	China	South-East Asia, East Asia & Oceania	3588	TRUST+TPPA	Cross-Sectional	Convenient	7	4	1	2
Liu, Y.,2018	China	South-East Asia, East Asia & Oceania	3588	TRUST+TPPA	Cohort(Baseline)	Rct	6	3	1	2
Liu, Z.,2017	China	South-East Asia, East Asia & Oceania	486	RPR+TPHA	Cross-Sectional	Convenient	8	4	2	2
Lobo, A. M.,2018	US	High Income	16797	ELISA+RPR+TPPA	Cohort(Baseline)	Convenient	7	3	1	3

						Sampling				
Lu, R.,2020	China	South-East Asia, East Asia & Oceania	4900	RPR+ELISA	Cross-Sectional	Convenient	8	4	1	3
Lucar, J.,2018	US	High Income	2652	nontreponemal test	Cohort(Baseline)	Convenient	8	3	2	3
Luo, H. B.,2012	China	South-East Asia, East Asia & Oceania	1118	PRP+TPPA	Cross-Sectional	Snow-Ball	7	3	1	3
Luo, Y.,2015	China	South-East Asia, East Asia & Oceania	259	RPR+TPHA	Cross-Sectional	RDS+Snowball+Convenient	7	2	2	3
Ma, Q.,2015	China	South-East Asia, East Asia & Oceania	424	RPR+ELISA	Cross-Sectional	RDS	6	3	0	3
Ma, W.,2016	China	South-East Asia, East Asia & Oceania	617	RPR+TPPA	Cross-Sectional	RDS	7	3	1	3
Maek-A-Nantawat, W.,2014	Thailand	South-East Asia, East Asia & Oceania	275	VDRL and Architect Syphilis TP	Cohort(Baseline)	Convenient	4	1	2	1
Mao, H.,2014	China	South-East Asia, East Asia & Oceania	511	RPR and TPPA	Cohort(Baseline)	Convenient	8	4	2	2
Mao, S. S.,2021	China	South-East Asia, East Asia & Oceania	1695	ELISA+TPPA	Cross-Sectional	Time-Location	6	3	0	3
Mao, X.,2018	China	South-East Asia, East Asia & Oceania	4496	RPR+TPPA	Cross-Sectional	Convenient+Snow-Ball	7	4	1	2
Marcel, M. S.,2018	Central African republic	Sub-saharan Africa	99	RPR+TPHA	Cross-Sectional	Convenient	6	2	1	3
Marcus, U.,2015	Germany	High Income	1380	TP antibody+cardiolipin	Cross-Sectional	Convenient	8	3	2	3
Martín-Sánchez, M.,2020	Australia	High Income	11317	RPR+TPPA	Cross-Sectional	Convenient	6	3	0	3
Mayer, K. H.,2012	US	High Income	365	VDRL/RPR+MHA/FTA	Cohort(Baseline)	Convenient	7	3	1	3

Mayer, K. H.,2012	US	High Income	850	RPR+fluorescent treponemal antibody absorbed test	Cross-Sectional	Convenient	6	3	0	3
Mebrahtu, H.,2018	UK	High Income	285018	not mentioned	Cross-Sectional	Survelliance	8	3	2	3
Mellouk, O.,2012	Morocco	North Africa & Middle East	669	serology	Cross-Sectional	RDS	7	3	1	3
Mizushima, D.,2018	Japan	High Income	861	RPR and TPHA	Cohort(Baseline)	Convenient	6	3	1	2
Mmbaga, E. J.,2017	Tanzania	Sub-saharan Africa	409	VDRL+TPHA	Cross-Sectional	RDS	7	4	1	2
Mmbaga, E. J.,2018	Tanzania	Sub-saharan Africa	646	VDRL+TPHA	Cross-Sectional	RDS	7	4	1	2
Mor, Z.,2012	Israel	High Income	1064	VDRL+TPHA/FTA	Cross-Sectional	Convenient	8	3	2	3
Morales Miranda, S.,2013	Guatemala	Latin America & Caribbean	433	not mentioned	Cross-Sectional	Convenient	7	3	1	3
Narayanan, P.,2013	India	South Asia	483	RPR+TPHA	Cross-Sectional	Convenient	8	4	1	3
Nash, J. L.,2014	Australia	High Income	37553	dark-ground microscopy, Treponema pallidum PCR, and serological tests (enzyme immunoassay, TPPA and RPR)	Cross-Sectional	Convenient Sampling	6	3	1	2
Nelson, L. E.,2019	Canada	High Income	86	EIA	Cross-Sectional	Non-Probability	8	4	1	3
Neto, P. L. F.,2021	Brazil	Latin America & Caribbean	123	VDRL+FTA	Cross-Sectional	Convenient	7	3	1	3
Nguyen, T.	Vietnam	South-East Asia, East	2467	RPR+TPHA	Cross-Sectional	Time-Location	4	2	0	2

V.,2016		Asia & Oceania								
Nguyen, T. V.,2021	Vietnam	South-East Asia, East Asia & Oceania	3087	RPR+TPHA	Cross-Sectional	National Survelliance	9	4	2	3
Ning, Z.,2018	China	South-East Asia, East Asia & Oceania	1120	Serology	Cross-Sectional	Convenient	6	3	1	2
Nishijima, T.,2016	Japan	High Income	885	RPR+TPHA	Cohort(Baseline)	Convenient	8	4	1	3
Nyombayire, J.,2016	Rwandan	Sub-saharan Africa	500	not mentioned	Cross-Sectional	RDS	5	2	1	2
Ooi, C.,2021	Australia	High Income	192	serology	Cross-Sectional	Convenient Sampling	6	3	1	2
Ouedraogo, H. G.,2019	Burkina Faso	Sub-saharan Africa	657	RPR+TP antibody	Cross-Sectional	RDS	6	3	0	3
Ozdemir, H. O.,2021	Turkey	North Africa & Middle East	159	CMIA+TPHA	Cross-Sectional	Convenient	7	3	1	3
Palacios, R.,2016	Spain	High Income	409	RPR+TP antibody	Cross-Sectional	Convenient Sampling	5	2	1	2
Palkar, A.,2019	India	South Asia	240	not mentioned	Cross-Sectional	Snow-Ball	5	2	1	2
Pan, X.,2015	China	South-East Asia, East Asia & Oceania	1316	ELISA, and TPPA/TRUST	Cross-Sectional	RDS	6	3	1	2
Park, J. N.,2013	Cameroon	Sub-saharan Africa	511	RPR+TPHA	Cross-Sectional	RDS	7	4	1	2
Parmley, L. E.,2022	Zimbabwe	Sub-saharan Africa	1176	Chembio Dual-Path Platform Syphilis Screen and Confirm Assay	Cross-Sectional	RDS	7	4	1	2
Pasvol, T.,2016	UK	High Income	4415	not mentioned	Cross-Sectional	Convenient	5	3	0	2
Pathela, P.,2022	US	High Income	741	serology	Cross-Sectional	Convenient Sampling	6	3	0	3
Perez-Brumer, A. G.,2013	Peru	Latin America & Caribbean	560	RPR+TPPA	Cross-Sectional	Convenient	7	3	1	3

Perkins, R.,2021	US	High Income	99	not mentioned	Cohort(Baseline)	Probability	7	3	1	3
Pham, Q. D.,2012	Vietnam	South-East Asia, East Asia & Oceania	380	TP antibody+TPHA	Cross-Sectional	Convenient	7	3	1	3
Phanuphak, N.,2018	Thailand	South-East Asia, East Asia & Oceania	137	RPR or VDRL+TPHA	Cohort(Baseline)	Probability	7	2	2	3
Philibert, P.,2014	France	High Income	116	TPHA and VDRL	Cross-Sectional	Convenient	6	3	1	2
Phillips, T. R.,2021	Australia	High Income	357	serology	Cross-Sectional	Convenient	7	4	1	2
Pitpitan, E. V.,2015	Mexico	Latin America & Caribbean	191	RPR+TPHA	Cross-Sectional	RDS	5	2	0	3
Pizzicato, L. N.,2017	Peru	Latin America & Caribbean	4374	RPR+TPHA	Cross-Sectional	Convenient Sampling	7	3	1	3
Prado, I.,2017	Brazil	Latin America & Caribbean	227	not mentioned	Cross-Sectional	Time-Location	9	4	2	3
Prado, I.,2017	Brazil	Latin America & Caribbean	338	not mentioned	Cross-Sectional	RDS	9	4	2	3
Price, H.,2013	UK	High Income	1114	EIA	Cross-Sectional	Convenient	7	4	1	2
Qi, J.,2015	China	South-East Asia, East Asia & Oceania	32701	RPR+TPPA	Cross-Sectional	Convenient	8	4	2	2
Qian, H. Z.,2014	China	South-East Asia, East Asia & Oceania	552	RPR+TPPA	Cross-Sectional	snow-ball	7	4	1	2
Qian, H.,2015	China	South-East Asia, East Asia & Oceania	3588	not mentioned	Cross-Sectional	Convenient	5	2	1	2
Qin, Q.,2016	China	South-East Asia, East Asia & Oceania	171311	ELISA+RPR	Cross-Sectional	Venue And Internet Based Sampling, Snowballing	7	3	1	3
Qu, L.,2016	China	South-East Asia, East Asia & Oceania	1611	ELISA+TURST	Cross-Sectional	Convenient Sampling	8	4	1	3
Ramakrishnan, L.,2015	India	South Asia	3739	PRP+TPPA	Cross-Sectional	Time-Location Cluster Sampling	8	4	1	3

Ramanathan, S.,2014	India	South Asia	1305	RPR+TPHA	Cross-Sectional	Convenient	7	4	1	2
Rawdah, W.,2015	UK	High Income	285	not mentioned	Cross-Sectional	Convenient Sampling	5	2	1	2
Read, P. J.,2013	Australia	High Income	98	fingerprick syphilis POC testing	Cross-Sectional	Convenient Sampling	5	2	1	2
Reback, C. J.,2018	US	High Income	286	RPR+FTA abs	Cohort(Baseline)	Convenient	7	3	1	3
Remis, R. S.,2016	Canada	High Income	437	CMIA+RPR+TPPA	Cross-Sectional	Convenient	7	3	1	3
Ribeiro, D.,2012	Brazil	Latin America & Caribbean	833	TP antibody IgM and IgG	Cross-Sectional	Stratified Cluster	6	2	1	3
Rice, C. E.,2016	US	High Income	235	RPR+TPPA/FTA-ABS	Cross-Sectional	Convenient Sampling	7	3	1	3
Ross, M. W.,2014	Tanzania	Sub-saharan Africa	262	TP Rapid Syphilis Assay	Cross-Sectional	RDS	6	3	1	2
Ruiseñor-Escudero, H.,2019	Togo	Sub-saharan Africa	683	TPHA+VDRL	Cross-Sectional	RDS	7	3	1	3
Safren, S. A.,2021	India	South Asia	608	RPR and TPHA	Cohort(Baseline)	Probability	6	3	1	2
Samarasekara, K.,2022	UK	High Income	6615	TPPA+ VDRL	Cross-Sectional	Convenient Sampling	6	3	1	2
Samji, H.,2022	Canada	High Income	9038	not mentioned	Cohort(Baseline)	Convenient	6	3	1	2
Sánchez-Gómez, A.,2015	Ecuador	Latin America & Caribbean	370	RPR+FTA-ABS	Cross-Sectional	RDS	8	4	2	2
Schumacher, C.,2019	US	High Income	230	not mentioned	Cohort(Baseline)	Convenient	8	3	2	3
Schumacher, C.,2020	US	High Income	230	not mentioned	Cohort(Baseline)	Convenient	7	4	1	2

Scott, H. M.,2015	US	High Income	1514	nontreponemal test+treponemal test	Cross-Sectional	Convenient	6	3	0	3
Semple, S. J.,2017	Mexico	Latin America & Caribbean	201	rapid testing	Cross-Sectional	RDS	7	3	1	3
Shah, N. S.,2014	El Salvador	Latin America & Caribbean	703	RPR+TPPA	Cross-Sectional	RDS	8	3	2	3
Shannon, C. L.,2019	US	High Income	641	TP antibody	Cross-Sectional	Convenient	7	3	1	3
She, M.,2012	China	South-East Asia, East Asia & Oceania	1693	RPR+TPPA	Cross-Sectional	Snow-Ball	7	4	1	2
She, M.,2013	China	South-East Asia, East Asia & Oceania	1693	RPR+TPPA	Cross-Sectional	Snowball Sampling	8	3	2	3
Shen, H.,2016	China	South-East Asia, East Asia & Oceania	2958	serology	Cross-Sectional	RDS And Snow-Ball	6	3	1	2
Shen, L.,2017	China	South-East Asia, East Asia & Oceania	657	RPR+TP antibody	Cross-Sectional	Convenient	7	4	1	2
Shiotsuka, M.,2016	Japan	High Income	723	RPR+TPLA	Cohort(Baseline)	Convenient	6	3	0	3
Silva, B. G. D.,2022	Brazil	Latin America & Caribbean	14959	not mentioned	Cross-Sectional	Convenient	6	3	1	2
Sirivongrangson, P.,2012	Thailand	South-East Asia, East Asia & Oceania	148	RPR+TPHA	Cross-Sectional	Convenient	6	3	1	2
Slurink, I. A.,2021	Netherlands	High Income	52149	nontrponemal+treponemal	Cross-Sectional	Surveillance Database	7	4	1	2
Smith, A. D.,2021	Kenya	Sub-saharan Africa	519	TPHA/ RPR	Cross-Sectional	RDS	6	3	1	2
Soares, C. C.,2014	Brazil	Latin America & Caribbean	588	TP rapid test	Cross-Sectional	RDS	9	4	2	3
Solangon, M.,2018	South Sudan	Sub-saharan Africa	152	not mentioned	Cross-Sectional	Purposive+Snow-Ball	5	2	1	2

Solomon, S. S.,2015	India	South Asia	12022	RPR and TPHA	Cross-Sectional	RDS	7	4	1	2
Spinner, C. D.,2018	Germany	High Income	296	TP serology	Cross-Sectional	Convenient	7	3	1	3
Sprenger, K.,2014	Switzerland	High Income	112	not mentioned	Cross-Sectional	Convenient	5	3	0	2
Storm, M.,	Nepal	South Asia	167	RPR+TPPA	Cross-Sectional	RDS	8	3	2	3
Streeck, H.,2022	Germany	High Income	1043	CMIA/TPPA/FTA+ RPR/VDRL	Cohort(Baseline)	Convenient	6	3	1	2
Subramanian, T.,2013	India	South Asia	4038	RPR+TPHA	Cross-Sectional	Convenient	7	4	1	2
Takano, M.,2020	Mongolia	Central Europe, Eastern Europe & Central Asia	849	TP antibody	Cohort(Baseline)	Convenient	6	3	0	3
Tang, W.,2015	China	South-East Asia, East Asia & Oceania	2618	RPR+TPPA	Cross-Sectional	Respondent-Driven And Snowball Sampling	7	3	1	3
Tang, W.,2015	China	South-East Asia, East Asia & Oceania	430	RPR+TPPA	Cohort(Baseline)	RDS	7	4	1	2
Tang, W.,2015	China	South-East Asia, East Asia & Oceania	410	RPR and TPPA	Cohort(Baseline)	RDS	7	3	1	3
Tomori, C.,2018	India	South Asia	11771	RPR+TPHA	Cross-Sectional	RDS	9	4	2	3
Tordoff, D. M.,2020	US	High Income	22761	darkfield microscopy and RPR+TPPA	Cohort(Baseline)	Convenient Sampling	7	3	1	3
Tu, W.,2022	China	South-East Asia, East Asia & Oceania	146	TRUST+TPPA	Cross-Sectional	Cluster Sampling	6	3	1	2
Tuan, N. A.,2020	Vietnam	South-East Asia, East Asia & Oceania	786	RPR+TPHA	Cross-Sectional	RDS	7	4	1	2
Van Griensven, F.,2013	Thailand	South-East Asia, East Asia & Oceania	1744	RPR+TP antibody	Cohort(Baseline)	Convenient	7	4	1	2

Verre, M. C.,2014	Peru	Latin America & Caribbean	2242	RPR+TPHA	Cross-Sectional	Convenient	6	3	1	2
Vilibić-Čavlek, T.,2018	Croatia	Central Europe, Eastern Europe & Central Asia	90	ELISA+WB	Cross-Sectional	Convenient	6	3	0	3
Wang, B.,2013	China	South-East Asia, East Asia & Oceania	307	ELISA+TPPA	Cross-Sectional	Convenient	6	2	1	3
Wang, H. Y.,2018	China	South-East Asia, East Asia & Oceania	476	RPR+TPPA	Cohort(Baseline)	Venue-Based+Peer Referrals	8	4	1	3
Wang, K.,2012	China	South-East Asia, East Asia & Oceania	2134	RPR+TPPA	Cross-Sectional	Snow-Ball	9	4	2	3
Wang, L.,2012	China	South-East Asia, East Asia & Oceania	37084	ELISA+RPR/TRUST	Cross-Sectional	Convenient+Snowball	7	4	1	2
Wang, L.,2020	China	South-East Asia, East Asia & Oceania	5000	TRUST+TPPA	Cross-Sectional	Snow-Ball	7	3	1	3
Wang, N.,2016	China	South-East Asia, East Asia & Oceania	#REF!	RPR+TPPA	Cross-Sectional	RDS	6	3	1	2
Wang, Q. Q.,2014	China	South-East Asia, East Asia & Oceania	622	TPPA and TRUST	Cohort(Baseline)	Snow-Ball Sampling	8	4	1	3
Wang, X.,2014	China	South-East Asia, East Asia & Oceania	6369	ELISA and RPR	Cross-Sectional	Convenient	6	3	1	2
Wang, X.,2017	China	South-East Asia, East Asia & Oceania	503	RPR+ELISA	Cohort(Baseline)	Convenient	7	3	1	3
Watson, D. L.,2019	US	High Income	606	TP antibody	Cross-Sectional	RDS	7	3	1	3
Wei, L.,2019	China	South-East Asia, East Asia & Oceania	3613	RPR+TPPA	Cross-Sectional	RDS+Convenient	7	3	1	3
Wei, S.,2013	China	South-East Asia, East Asia & Oceania	600	RPR+TPPA	Cross-Sectional	Snow-Ball Sampling	9	4	2	3
Weng, R. X.,2019	China	South-East Asia, East	5966	TRUST+TPPA	Cross-Sectional	Convenient	7	3	1	3

		Asia & Oceania				Sampling				
Wesselmann, J.,2018	Germany	High Income	859	VDRL+TPPA	Cohort(Baseline)	Convenient	8	3	2	3
Wheldon, C. W.,2022	US	High Income	395	not mentioned	Cohort(Baseline)	Convenient	5	3	0	2
Willekens, R.,2021	Spain	High Income	301	not mentioned	Cross-Sectional	Convenient	5	3	1	1
Wirtz, A. L.,2013	Malawi	Sub-saharan Africa	337	TPHA+Confirmatory	Cross-Sectional	RDS	8	4	2	2
Wu, H.,2012	Taiwan	South-East Asia, East Asia & Oceania	5924	RPR+TPHA	Cross-Sectional	Convenient	8	3	2	3
Wu, H.,2019	China	South-East Asia, East Asia & Oceania	447	TRUST+TP antibody	Case-Control	Convenient	7	4	0	3
Wu, J.,2016	China	South-East Asia, East Asia & Oceania	522	ICE+TPPA	Cross-Sectional	Convenient	7	4	1	2
Wu, Q.,2015	China	South-East Asia, East Asia & Oceania	4904	RPR+TPPA	Cross-Sectional	Convenient	9	4	2	3
Wu, Z. L.,2018	China	South-East Asia, East Asia & Oceania	2800	RPR+ELISA	Cross-Sectional	Snowball Sampling	7	3	1	3
Wu, Z.,2013	China	South-East Asia, East Asia & Oceania	47231	RPR/TRUST+TPPA	Cross-Sectional	Snow-Ball Sampling+RDS	9	4	2	3
Xie, N.,2022	China	South-East Asia, East Asia & Oceania	220	RPR+CMIA	Cohort(Baseline)	Convenient	7	3	1	3
Xu, J. J.,2014	China	South-East Asia, East Asia & Oceania	625	RPR+TPPA	Cross-Sectional	Snowball	8	3	2	3
Xu, J. J.,2014	China	South-East Asia, East Asia & Oceania	3830	RPR+TPPA	Cross-Sectional	Convenient	8	3	2	3
Xu, J. J.,2016	China	South-East Asia, East Asia & Oceania	4496	RPR and TPPA	Cross-Sectional	Convenient+Snow-Ball	7	4	1	2
Xu, J. J.,2017	China	South-East Asia, East	4496	RPR+TPPA	Cross-Sectional	Internet+Venue-	6	3	0	3

		Asia & Oceania				Based+Rds				
Xu, J.,2018	China	South-East Asia, East Asia & Oceania	686	RPR+TPPA	Cohort(Baseline)	Internet+Venue Based+Chain Referral	8	3	2	3
Xu, Y. Y.,2016	China	South-East Asia, East Asia & Oceania	3320	TRUST+TP antibody	Cross-Sectional	Convenient+Snow-Ball	7	4	1	2
Xu, Y.,2022	China	South-East Asia, East Asia & Oceania	775	RPR+TPPA	Cross-Sectional	Snowball+Convenie nt	8	3	2	3
Yan, G.,2022	China	South-East Asia, East Asia & Oceania	9218	ELISA+RPR	Cross-Sectional	Snow-Ball Sampling	6	3	0	3
Yan, H.,2016	China	South-East Asia, East Asia & Oceania	1019	RPR+TPPA	Cross-Sectional	RDS	7	4	1	2
Yang, H. T.,2014	China	South-East Asia, East Asia & Oceania	839	ELISA+confirmed	Cohort(Baseline)	Clustering	6	3	1	2
Ye, Z. H.,2022	China	South-East Asia, East Asia & Oceania	177	RPR+TPPA	Cross-Sectional	Convenient	6	2	1	3
Yu, Y. Q.,2016	China	South-East Asia, East Asia & Oceania	4496	TP antibody	Cross-Sectional	Convenient	7	4	1	2
Zalla, L. C.,2019	Haiti	Latin America & Caribbean	520	SD Bioline Syphilis 3.0	Cross-Sectional	Stratified Random Sampling	8	4	1	3
Zeng, G.,2014	China	South-East Asia, East Asia & Oceania	2331	ELISA+TURST	Cross-Sectional	Snowball Method, And Using Online Recruitment.	7	3	1	3
Zeng, Y.,2014	China	South-East Asia, East Asia & Oceania	567	not mentioned	Cross-Sectional	Stratified Snowball	8	3	2	3
Zhang, C.,2016	China	South-East Asia, East Asia & Oceania	3588	TRUST+TPPA	Cross-Sectional	Convenient	6	3	0	3
Zhang, C.,2020	China	South-East Asia, East Asia & Oceania	256	TP antibody	Cross-Sectional	Convenient	8	4	2	2
Zhang, D.	China	South-East Asia, East	408	TRUST+ELISA	Cross-Sectional	Convenient	7	4	1	2

Y.,2014		Asia & Oceania								
Zhang, H.,2016	China	South-East Asia, East Asia & Oceania	3588	TRUST+TPPA	Cohort(Baseline)	Convenient	7	3	1	3
Zhang, J.,2018	China	South-East Asia, East Asia & Oceania	646	RPR+TPPA	Cohort(Baseline)	Convenient	7	3	1	3
Zhang, L.,2012	China	South-East Asia, East Asia & Oceania	503	RPR+TPPA	Cross-Sectional	RDS	8	3	2	3
Zhang, L.,2013	China	South-East Asia, East Asia & Oceania	463	RPR+TPPA	Cross-Sectional	Snow-Ball	8	3	2	3
Zhang, L.,2021	China	South-East Asia, East Asia & Oceania	4813	RPR+TP antibody	Cross-Sectional	Snow Ball	7	3	1	3
Zhang, S. H.,2016	China	South-East Asia, East Asia & Oceania	427	not mentioned	Cross-Sectional	Snow-Ball	7	3	1	3
Zhang, T.,2013	China	South-East Asia, East Asia & Oceania	208	RPR+TPHA	Cross-Sectional	Venue-Based+Snow-Ball	6	2	1	3
Zhang, X.,2013	China	South-East Asia, East Asia & Oceania	302	RPR+TP antibody	Cross-Sectional	Snow-Ball	6	3	0	3
Zhang, X.,2017	China	South-East Asia, East Asia & Oceania	300	TRUST+TPPA	Cross-Sectional	Convenient	7	3	1	3
Zhang, Y.,2012	China	South-East Asia, East Asia & Oceania	3314	RPR+TPPA	Cross-Sectional	Stratified Snowball	6	3	0	3
Zhang, Y.,2019	China	South-East Asia, East Asia & Oceania	6568	RPR+TRUST	Cross-Sectional	Snowball	7	3	1	3
Zhao, D. S.,2018	China	South-East Asia, East Asia & Oceania	266	RPR+ELISA	Cross-Sectional	RDS	6	3	0	3
Zhao, J.,2012	China	South-East Asia, East Asia & Oceania	1651	PRP+TPPA	Cross-Sectional	Time-Location Sampling (Tls)	7	3	1	3
Zhao, J.,2014	China	South-East Asia, East Asia & Oceania	801	RPR+TPPA	Cross-Sectional	Convenient	9	4	2	3
Zhao, J.,2015	China	South-East Asia, East	1154	PRP+TPPA	Cross-Sectional	RDS+Time	7	3	1	3

		Asia & Oceania				Location Sampling				
Zhao, N.,2019	China	South-East Asia, East Asia & Oceania	183	RPR+TPPA	Cross-Sectional	Convenient	6	2	1	3
Zhao, P.,2021	China	South-East Asia, East Asia & Oceania	2184	RPR+TPPA	Cross-Sectional	Sentinel Surveillance	7	3	1	3
Zhao, P.,2022	China	South-East Asia, East Asia & Oceania	174	self-testing	Cross-Sectional	Convenient Sampling	6	3	0	3
Zhao, Y.,2015	China	South-East Asia, East Asia & Oceania	1312	not mentioned	Cross-Sectional	Snow-Ball	7	4	1	2
Zheng, C.,2016	China	South-East Asia, East Asia & Oceania	3717	PRP+TPPA	Cross-Sectional	Convenient Sampling+RDS	8	4	1	3
Zheng, J.,2012	China	South-East Asia, East Asia & Oceania	157	RPR+TPPA	Cross-Sectional	Convenient	7	3	1	3
Zhong, F.,2014	China	South-East Asia, East Asia & Oceania	2603	RPR+TPPA	Cross-Sectional	Snow-Ball	7	4	1	2
Zhong, F.,2016	China	South-East Asia, East Asia & Oceania	178	Rapid Test for Antibody+confirmation testing	Cross-Sectional	Convenient	6	2	2	2
Zhong, F.,2017	China	South-East Asia, East Asia & Oceania	178	TP antibody	Cross-Sectional	Convenient Sampling	7	3	1	3
Zhu, Z.,2019	China	South-East Asia, East Asia & Oceania	3031	RPR+TPPA	Cross-Sectional	Snowball+Convenient	8	3	2	3
Zohrabyan, L.,2013	Republic of Moldova	Central Europe, Eastern Europe & Central Asia	397	TPHA IgG and IgM	Cross-Sectional	RDS	7	4	1	2
Zou, H.,2013	Australia	High Income	4758	electrical medical record	Cross-Sectional	Convenient	5	3	1	1

Table S2. Characteristics of the included incidence studies.

Author, Year	Country	Region	Sample size	sy testing	Study design	Sampling methods	Study Quality	Selection	Comparability	Outcome
Allan-Blitz, L. T.,2021	US	High Income	1180	RPR+TPPA	Cohort(Follow Up)	Convenient	8	3	2	3
Allen, H.,2018	UK	High Income	112.96	not mentioned	Cohort(Follow Up)	Survelliance	5	3	1	1
Allen, H.,2021	UK	High Income	112960	not mentioned	Cohort(Follow Up)	Convenient Sampling	7	3	1	2
Ananworanich J,2013	Thailand	South-East Asia, East Asia & Oceania	2106	RPR+immunochromatography	Cohort(Follow Up)	Convenient	8	4	1	3
Ang, L. W.,2020	Singapore	High Income	2477	RPR or VDRL	Cohort(Follow Up)	Surveillance Database	7	4	1	2
Apers, L.,2013	Belgium	High Income	1315.8	RPR+TPPA	Cohort(Follow Up)	Convenient	8	4	1	3
Ayerdi, O.,2021	Spain	High Income	207.74	RPR, EIA, and TPPA	Cohort(Follow Up)	Convenient	6	3	1	2
Bissio, E.,2017	Argentina	High Income	1150	VDRL and medical history	Cohort(Follow Up)	Convenient	7	2	2	3
Burchell, A. N.,2015	Canada	High Income	4497.5	CMIA+RPR	Cohort(Follow Up)	Convenient	6	4	1	1
Chu, Z. X.,2018	China	South-East Asia, East Asia & Oceania	568.5	RPR+TPPA	Cohort(Follow Up)	Snowball	6	3	0	3
Cielniak, I.,2019	Poland	Central Europe, Eastern Europe & Central Asia	51.5	not mentioned	Cohort(Follow Up)	Convenient Sampling	6	3	1	2
Colby, D. J.,2015	Thailand	South-East Asia, East Asia & Oceania	278.26	VDRL+TPHA	Cohort(Follow Up)	Convenient	6	3	1	2
Coll, J.,2018	Spain	High Income	494	nontrponemal+treponemal	Cohort(Follow Up)	Convenient	6	3	1	2
Cope, A.	US	High Income	493.8	RPR+MHA-TP	Cohort(Follow Up)	Convenient	6	2	1	3

B.,2014										
Dong, Z.,2014	China	South-East Asia, East Asia & Oceania	1106.67	RPR+TPPA	Cohort(Follow Up)	Snowball	9	4	2	3
Dramé, F. M.,2013	Senegal	Sub-saharan Africa	61	RPR+TP antibody	Cohort(Follow Up)	Convenient	5	1	1	3
Farfour, E.,2017	France	High Income	1279	RPR+TPHA	Cohort(Follow Up)	Convenient	7	4	1	2
Fata, L. L.,2017	France	High Income	97	serology	Cohort(Follow Up)	Convenient	6	3	1	2
Ganley, K. Y.,2021	Mexico	Latin America & Caribbean	161.04	VDRL+TP antibody	Cohort(Follow Up)	Convenient	8	4	1	3
Goddard, S. L.,2019	Australia	High Income	1406.68	EIA+TPPA/FTA+RPR	Cohort(Follow Up)	Convenient	6	2	1	3
Gorbach, P. M.,2019	US	High Income	1638.4	serology	Cohort(Follow Up)	Convenient Sampling	7	2	1	3
Gravett, R. M.,2020	US	High Income	78.5	RPR+TPPA	Cohort(Follow Up)	Convenient	6	2	1	3
Hart-Malloy, R.,2019	US	High Income	14770.8	not mentioned	Cohort(Follow Up)	Convenient	7	3	1	3
Hazra, A.,2019	US	High Income	14858	RPR	Cohort(Follow Up)	Convenient	7	4	1	2
Hoornenborg, E.,2019	Netherlands	High Income	681.7	LIAISON Treponema Screen	Cohort(Follow Up)	Convenient	8	3	2	3
Hu, Q.,2014	China	South-East Asia, East Asia & Oceania	570	RPR+TPPA	Cohort(Follow Up)	Convenient	9	4	2	3
Huang, Y. F.,2013	Taiwan	South-East Asia, East Asia & Oceania	665.5		Cohort(Follow Up)	Convenient	4	2	0	2
Jansen, K.,2015	Germany	High Income	4057	CLIA+TPPA+FTA-Abs/RPR	Cohort(Follow Up)	Convenient	6	3	1	2
Jia, Z.,2015	China	South-East Asia, East Asia & Oceania	5099	RPR+TPPA	Cohort(Follow Up)	Convenient	7	3	1	3

Jin, F.,2012	Australia	High Income	2667	serology	Cohort(Follow Up)	Convenient	7	3	1	3
Karkashadze, E.,2019	US	High Income	38.3	not mentioned	Cohort(Follow Up)	Convenient	7	3	1	3
Kawi, N. H.,2022	Indonesia	South-East Asia, East Asia & Oceania	488.4	RPR+rapid TP antibody	Cohort(Follow Up)	Convenient	9	4	2	3
Kelley, C. F.,2015	US	High Income	837.7	RPR+TP antibody	Cohort(Follow Up)	Time-Space Venue	7	3	1	3
Lachowsky, N. J.,2016	Canada	High Income	1075.3	biological specimen or self-reported	Cohort(Follow Up)	RDS	6	3	1	2
Li, D.,2012	China	South-East Asia, East Asia & Oceania	506.06	RPR+TPPA	Cohort(Follow Up)	Convenient	7	3	1	3
Li, D.,2016	China	South-East Asia, East Asia & Oceania	561.06	RPR+TPPA	Cohort(Follow Up)	Convenient	8	4	1	3
Liu, A. Y.,2016	US	High Income	481	VDRL/RPR+FTA	Cohort(Follow Up)	Convenient	7	3	1	3
Liu, G.,2015	China	South-East Asia, East Asia & Oceania	1016.4	RPR+TPPA	Cohort(Follow Up)	RDS	9	4	2	3
Mao, H.,2014	China	South-East Asia, East Asia & Oceania	152.95	RPR and TPPA	Cohort(Follow Up)	Convenient	9	4	2	3
Mathur, P.,2014	US	High Income	2059.2	RPR+confirmatory treponemal test	Cohort(Follow Up)	Convenient	7	2	2	3
Mayer, K. H.,2012	US	High Income	180	VDRL/RPR+MHA/FTA	Cohort(Follow Up)	Convenient	8	4	1	3
Menza, T. W.,2022	US	High Income	17433	RPR	Cohort(Follow Up)	Convenient	6	3	0	3
Mizushima, D.,2018	Japan	High Income	71.6	RPR and TPHA	Cohort(Follow Up)	Convenient	6	3	1	2
Molina, J. M.,2018	France	High Income	163.1	VDRL+TPHA	Cohort(Follow Up)	Probability	8	4	1	3
Montanõ, M. A.,2020	Peru	Latin America & Caribbean	77.3	RPR+TPHA	Cohort(Follow Up)	Probability	7	4	0	3

Nguyen, V. K.,2018	Canada	High Income	218	RPR+TPPA	Cohort(Follow Up)	Convenient	8	4	1	3
Nishijima, T.,2016	Japan	High Income	2562	RPR+TPHA	Cohort(Follow Up)	Convenient	8	4	1	3
Novak, R. M.,2018	US	High Income	21240	RPR	Cohort(Follow Up)	Convenient	8	3	2	3
Park, H.,2016	Peru	Latin America & Caribbean	1388.9	RPR+TPPA	Cohort(Follow Up)	Convenient	7	2	2	3
Peel, J.,2021	Australia	High Income	800	RPR+ELISA+TPPA	Cohort(Follow Up)	Convenient Sampling	7	3	1	2
Putot, A.,2017	French Antilles	Latin America & Caribbean	2384.3	VDRL+TPHA	Cohort(Follow Up)	Convenient	8	3	2	3
Reback, C. J.,2018	US	High Income	186.5	RPR+FTA abs	Cohort(Follow Up)	Convenient	7	4	1	2
Roth, J. A.,2020	Switzerland	High Income	17653.1	VDRL or RPR+TPPA/TPHA or CLIA or CMIA or IgG/IgM immunoassy	Cohort(Follow Up)	Probability	9	4	2	3
Saxton, P. J. W.,2022	New Zealand	High Income	133.25	not mentioned	Cohort(Follow Up)	Convenient Sampling	8	4	1	2
Schumacher, C.,2020	US	High Income	7179.8	nontreponemal test	Cohort(Follow Up)	Convenient	8	4	1	3
Takano, M.,2020	Mongolia	Central Europe, Eastern Europe & Central Asia	937.1	TP antibody	Cohort(Follow Up)	Convenient	6	3	0	3
Tan, D. H. S.,2018	Canada	High Income	48.78	serology	Cohort(Follow Up)	Convenient Sampling	6	3	1	2
Tang, W.,2015	China	South-East Asia, East Asia & Oceania	315.6	RPR+TPPA	Cohort(Follow Up)	RDS	7	3	1	2

Thienkrua, W.,2016	Thailand	South-East Asia, East Asia & Oceania	3351	RPR+TP antibody	Cohort(Follow Up)	Convenient	7	3	1	3
Traeger, M. W.,2019	Australia	High Income	3185	not mentioned	Cohort(Follow Up)	Convenient Sampling	7	2	1	3
Traeger, M. W.,2022	Australia	High Income	21978.9	not mentioned	Cohort(Follow Up)	Surveillance	6	2	1	3
Van Bilsen, W. P. H.,2020	Netherlands	High Income	4966	RPR+FTA-ABS	Cohort(Follow Up)	Convenient Sampling	7	3	1	2
Wang, Q. Q.,2014	China	South-East Asia, East Asia & Oceania	302	TPPA and TRUST	Cohort(Follow Up)	Snow-Ball Sampling	8	4	1	3
Wang, Y.,2015	China	South-East Asia, East Asia & Oceania	535.5	TRUST+TPPA	Cohort(Follow Up)	RDS+Snow Ball	9	4	2	3
Xie, N.,2022	China	South-East Asia, East Asia & Oceania	166	RPR+CMIA	Cohort(Follow Up)	Convenient	7	4	1	2
Xu, J.,2013	China	South-East Asia, East Asia & Oceania	279.1	RPR+TPPA	Cohort(Follow Up)	Convenient	8	3	2	3
Xu, J.,2018	China	South-East Asia, East Asia & Oceania	332	RPR+TPPA	Cohort(Follow Up)	Internet+Venue Based+Chain Referral	8	3	2	3
Yang, H. T.,2014	China	South-East Asia, East Asia & Oceania	297.8	ELISA+confirmed	Cohort(Follow Up)	Clustering	6	3	0	3
Zeggagh, J.,2020	France	High Income	269.2	serology	Cohort(Follow Up)	Convenient	7	4	1	2
Zhang, F.,2021	China	South-East Asia, East Asia & Oceania	219.3	not mentioned	Cohort(Follow Up)	Convenient	8	3	2	3
Zhou, N.,2018	China	South-East Asia, East Asia & Oceania	1959.94	RPR+ELISA	Cohort(Follow Up)	Convenient	8	3	2	3

Table S3. Estimates of syphilis incidence by HIV status

Geographic region	HIV+ MSM			HIV- MSM		
	Record	Follow up person year	Pooled incidence estimates, /1000 py (95% CI)	Record	Follow up person year	Pooled incidence estimates, /1000 py (95% CI)
**South-East Asia, East Asia & Oceania**	**3**	**1513.8**	**170.2 (120.4-226.7)**	**6**	**8565.6**	**69.3 (54.7-85.1)**
China	1	570.0	194.7 (163.2-228.3)	6	8565.6	69.3 (54.7-85.1)
Taiwan, *province of China*	1	665.5	199.8 (170.3-231.1)			
Thailand	1	278.3	115.0 (80.0-155.3)			
**High Income**	**19**	**210133.3**	**68.8 (37.0-109.4)**	**12**	**7104.2**	**84.1 (40.4-141.3)**
Argentina	1	1150.0	148.7 (128.7-169.9)			
Australia	1	505.0	91.1 (67.4-117.9)	3	4368.7	33.6 (2.7-95.4)
Belgium	1	1315.8	133.0(115.2-151.9)			
Canada	2	5258.4	19.6 (16.0-23.5)	2	366.0	71.0 (46.0-100.6)
France	1	1279.0	43.0 (32.5-54.9)	2	432.3	225.9 (6.7-613.3)
Germany	1	4057.0	40.7 (34.8-47.0)			
Netherlands				1	681.7	110.0 (87.6-134.7)
New Zealand				1	133.3	105.1 (58.0-163.5)
Singapore	1	2477.0	90.0 (79.1-101.6)			
Spain				1	207.7	154.0 (107.9-206.6)
Switzerland	1	17653.1	490.0 (482.6-497.4)			
UK	2	113073.0	30.5 (29.4-31.5)			
US	8	63365.0	48.8 (21.0-86.8)	2	914.5	24.9 (15.2-36.7)
**Latin America & Caribbean**	**2**	**2461.6**	**53.0 (44.2-62.5)**			
French Antilles	1	2384.3	54.9 (46.1-64.5)			
Peru	1	77.3	77.6 (26.6-149.5)			
**Total**	**24**	**214108.7**	**150.1 (115.4-209.7)**	**18**	**15669.8**	**79.5 (49.6-115.4)**

HIV, human immunodeficiency virus; MSM, men who have sex with men; CI, confidence interval.

Bold values represent results at the global or regional level, and non-bold values represent results at national level.
